# Survey of solution dynamics in Src kinase reveals allosteric cross talk between the ligand binding and regulatory sites

**DOI:** 10.1038/s41467-017-02240-6

**Published:** 2017-12-18

**Authors:** Michael Tong, Jeff G. Pelton, Michelle L. Gill, Weibing Zhang, Francis Picart, Markus A. Seeliger

**Affiliations:** 1 Department of Pharmacological Sciences, Stony Brook University Medical School, Stony Brook, NY 11794 USA; 2 QB3 Institute, University of California, Berkeley, CA 94720 USA; 3 Structural Biophysics Laboratory, National Cancer Institute, Frederick, MD 21702 USA; 4 Department of Chemistry, Stony Brook University, Stony Brook, NY 11790 USA

## Abstract

The catalytic domain of protein tyrosine kinases can interconvert between active and inactive conformations in response to regulatory inputs. We recently demonstrated that Src kinase features an allosteric network that couples substrate-binding sites. However, the extent of conformational and dynamic changes that are propagated throughout the kinase domain remains poorly understood. Here, we monitor by NMR the effect of conformationally selective inhibitors on kinase backbone dynamics. We find that inhibitor binding and activation loop autophosphorylation induces dynamic changes across the entire kinase. We identify a highly conserved amino acid, Gly449, that is necessary for Src activation. Finally, we show for the first time how the SH3–SH2 domains perturb the dynamics of the kinase domain in the context of the full length protein. We provide experimental support for long-range communication in Src kinase that leads to the relative stabilization of active or inactive conformations and modulation of substrate affinity.

## Abstract (web summary)

Src is a prototypical signaling non-receptor protein tyrosine kinase that interconverts between distinct conformations. Here the authors use variants of the kinase-inhibitor dasatinib to define three specific conformational states of the Src kinase and shed insight on the effect of conformation-specific inhibitors on Src dynamics.

## Introduction

**Fig. 1:**
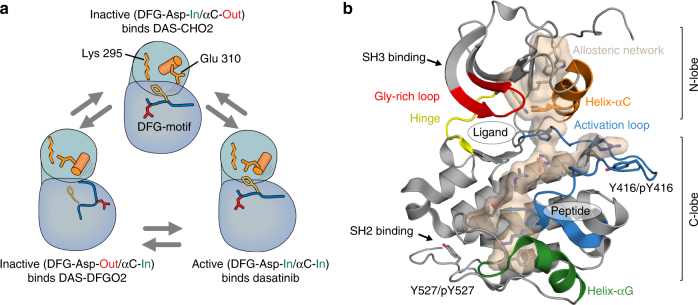
Src can adopt distinct conformations and integrates diverse inputs that regulate its kinase activity. **a** Distinct active and inactive conformations are induced when SrcKD binds to conformation-selective ligands. Dasatinib stabilizes the “DFG-Asp-in/αC-in” active conformation, whereas DAS-DFGO2, and DAS-CHO2, stabilize the “DFG-Asp-out/αC-in” and “DFG-Asp-in/αC-out” inactive conformations, respectively. **b** In Src, allosteric communication between the ATP-, substrate peptide-binding sites and the regulatory sites is propagated through an allosteric network (stick and surface representation) that was previously identified from MD simulations^19^. This network spans the kinase domain from the N-lobe to the C-lobe and includes catalytic and regulatory elements, and suggests a mechanism for integrating diverse regulatory input signals

Protein tyrosine kinases (PTKs) are important mediators of signal transduction because they control many different cellular processes such as cell proliferation, adhesion, and motility^1^. Therefore, tightly regulated PTK activity is critical for normal cell function. Conversely, loss of kinase regulation can cause diseases including cancer^2,3^. Src, is a prototypical non-receptor PTK, that comprises a catalytic kinase domain (referred to as SrcKD), two regulatory domains (SH3, SH2), a unique domain, and an amino-terminal membrane-localizing tail (SH4)^1,4^. The active conformation of SrcKD is defined by two hallmarks: (i) a salt-bridge between the catalytic lysine, Lys295 (chicken Src numbering), and Glu310 in helix-αC (“αC-in” conformation), and (ii) Asp404 in the Asp-Phe-Gly (DFG) motif at the amino-terminus of the activation loop (residues 404–432) facing into the active site to coordinate Mg^2+^•ATP (“DFG-Asp-in” conformation) (Fig. 1a). Disruption of either hallmark inactivates the kinase: outward rotation of helix-αC breaks the Lys295-Glu310 salt-bridge (“DFG-Asp-in/αC-out” conformation), and rotation of the DFG motif leaves Asp404 facing into the solvent (“DFG-Asp-out/αC-in” conformation). Other regulatory elements in the active site include the glycine-rich P-loop (residues 273–282, also referred to as Phosphate-binding or P-loop), and the hinge (residues 339–345), which connects the amino-terminal N-lobe with the carboxy-terminal C-lobe (Fig. 1b). The hinge regulates inter-lobe motions and increased hinge dynamics following activation loop autophosphorylation, has been implicated in activating catalysis^5^. The inherent conformational plasticity and dynamic properties of the kinase domain allows Src to switch between an ensemble of inactive and active conformations. For example, MD simulations of SrcKD undergoing a transition from an inactive to active-like open state, indicate that conformational changes occur around the active site^6^. During this transition the unphosphorylated activation loop unfolds as SrcKD intermediate states were sampled along the pathway^6^. These events were also coupled with the switching of electrostatic interactions between catalytic and regulatory elements around the active site, and assembly of the hydrophobic R-spine^6^, a dynamic element important in stabilizing the active conformation^7^.

Regulatory signals can shift the populations of active and inactive conformations (Fig. 1b). For example, in full length Src binding of the SH3 domain to a polyproline linker and binding of the SH2 domain to the Tyr527-phopshorylated carboxy-terminal tail stabilizes the autoinhibited assembled state^4,8^. In contrast, activation loop autophosphorylation at Tyr416, carboxy-terminal tail dephosphorylation (Tyr527), binding of cognate peptides to the SH3 and SH2 domains can stabilize the active disassembled state^8–10^. Interestingly, if autophosphorylation at Tyr416 precedes phosphorylation at Tyr527, then the active state persists and can override the autoinhibitory effects of phospho-Tyr527^11^. SrcKD activity is thus dependent on the integration of diverse input signals. Because these signals originate from distinct sites, signal propagation is necessary between the regulatory sites and the catalytic ATP- and substrate-binding sites. This suggests that allosteric communication plays an important role for the regulation of Src kinase.

Allostery is defined as the process by which biological molecules transmit the effect of binding at one site to another, allowing for regulation of activity^12^. The KNF (Koshland–Nemethy–Filmer) and MWC (Monod–Wyman–Changeux) paradigms^13,14^ describe allostery and co-operative binding based on conformational changes between well-defined structural states but did not take into account factors such as conformational dynamics, monomeric states, disordered proteins, and proteins with negligible conformational changes^15^. The current population-shift paradigm accounts for these factors by considering proteins as conformational ensembles^12,16^.

The general mechanism behind allostery in Src has been investigated previously but was not completely elucidated^17,18^. Recently, we have unraveled more details about the mechanism in Src by showing that allosteric communication is mediated through a network (Fig. 1b) of dynamically coupled residues that spans SrcKD^19^. MD simulations and experiments showed that the network couples the ATP- and substrate peptide-binding sites and mediates cooperativity between them^19^. Interestingly, the nature of cooperativity in Src differs from that of PKA, a model Ser/Thr kinase. Although PKA binds substrates with positive cooperativity^20^, Src binds substrates with negative cooperativity and products with positive cooperativity^19^. This suggests a distinct allosteric mechanism between Tyr and Ser/Thr kinases. Since the allosteric network spans SrcKD, it suggests that long-range crosstalk can propagate beyond the ATP- and substrate peptide-binding sites and affect its kinase activity. Such long-range effects have been alluded to in various computational studies that have described long-range coupling^6^, an increase in the kinase active population upon activation loop autophosphorylation^21^, and crosstalk between SrcKD and its SH3 and SH2 domains^22^. In addition, binding of conformationally selective ATP-competitors can allosterically regulate the protein–protein interactions of kinases. For example, ATP-competitive inhibitors can modulate interactions of MAPKs with upstream activators and phosphatases, and ATP-competitive inhibitors of Ire1α kinase can control kinase dimerization and activity of its RNase domain^23,24^.

**Fig. 2:**
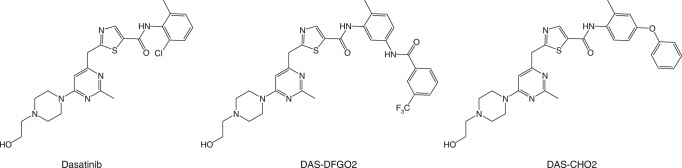
Chemical structure of dasatinib, DAS-DFGO2 and DAS-CHO2. The ligands feature a common dasatinib scaffold with substituents at the phenyl moiety^30^

Crystal structures of SrcKD bound to conformationally selective ATP-competitive inhibitors often differ only in structural details and crystallographic B-factors near the inhibitor binding site, they are typically identical in the C-lobes of kinases (Supplementary Fig. 1). However, allosteric regulation can occur without conformational changes through dynamic changes^25,26^. We therefore study in Src ligand-mediated long-range allosteric effects and how they modulate the accessibility of the regulatory SH3–SH2 domains by solution NMR methods^27^. Because SrcKD can adopt distinct active and inactive conformations^28,29^, we expect that stabilizing certain conformations through conformationally selective ligands will show its distinct dynamic signatures^27^. For example, dasatinib, binds to the active conformation (Fig. 1a), but chemical substitutions on the dasatinib scaffold (Fig. 2) yield variants that instead target distinct inactive conformations (Fig. 1a) without compromising potency (Supplementary Table 1)^30^.

In this study, we survey a panel of different conformation-selective ligands (Fig. 2) and their effects on SrcKD backbone conformation and dynamics, using chemical shift perturbations (CSPs), and resonance intensity perturbations (IPs), respectively. Our results suggest that ligand binding induces long-range perturbations that modulate the dynamics of regulatory sites. We extended this investigation further, by comparing how activation loop autophosphorylation followed by binding of dasatinib and substrate peptide affect the conformation and dynamics of SrcKD. We find that these active states show distinct conformational and dynamic differences, suggesting long-range perturbations to regulatory sites. We further investigate the underlying dynamic processes for the activation loop autophosphorylated and dasatinib-bound SrcKD state (pSrcKD•dasatinib) by fast backbone dynamics experiments. Our results reveal that fast internal motions dominate but also suggest the presence of conformational exchange. In addition, activation loop autophosphorylation results in structural and dynamic changes at the conserved residue Gly449 located within the regulatory helix-αF. We find that Gly449 mediates autophosphorylation-dependent maximal kinase activity in full length Src (defined as Src3D minus the unique and SH4 domains). Using a novel isotopic labeling and protein ligation strategy, we were able to extend our NMR backbone structural and dynamic analysis for the first time to the 52 kDa full length Src. We find that the regulatory SH3–SH2 domains can perturb the kinase domain suggesting transiently assembled and disassembled states.

## Results

### Backbone assignments of distinct SrcKD states

To address how different ligands affect the backbone conformation and dynamics of SrcKD, we assigned backbone amide resonances for four distinct states (apo SrcKD 75% assigned, SrcKD•dasatinib 89%, SrcKD•DAS-DFGO2 85%, and SrcKD•DAS-CHO2 74%). The latter two ligands are based on the chemical scaffold of dasatinib (Fig. 2) with chemical modifications at the phenyl meta-, and para-position. These ligands bind selectively to the “DFG-Asp-out/αC-in” (DAS-DFGO2), and the “DFG-Asp-in/αC-out” (DAS-CHO2), inactive conformations (Fig. 1a)^30^. In contrast, dasatinib binds to the “DFG-Asp-in/αC-in” active conformation (Fig. 1a)^31^. These ligands were added in excess to obtain SrcKD in defined active/inactive conformations and to prevent flipping between the “DFG-Asp-out and Asp-in” conformations. As all ligands (Supplementary Table 1) have similar affinities (*K*_d_ ranging from 0.07 to 2.7 nM)^30^ and dissociation rate constants (*k*_off_ ranging from 1.8 to 8.6 × 10^−4^ s^−1^)^30^, we do not expect that differences in their binding kinetics affect the observable protein dynamics.

### Conformation-selective ligands induce few long-range CSPs

**Fig. 3:**
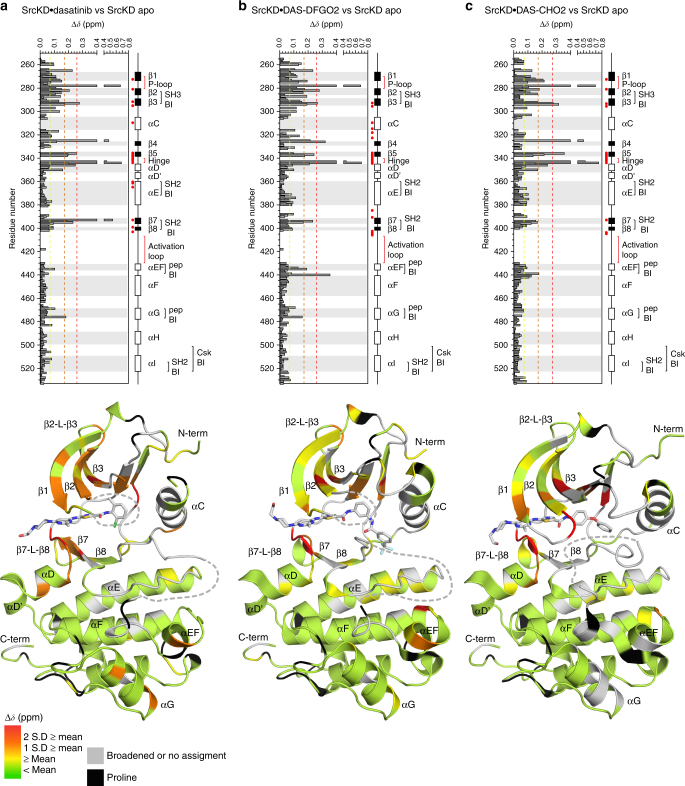
Binding of conformation-selective ligands to apo SrcKD induces CSP near the ATP-binding site and at select C-lobe residues. CSPs induced in SrcKD upon binding: **a** dasatinib, **b** DAS-DFGO2, **c** DAS-CHO2, were analyzed by histograms and by CSP mapping to their structures (PDB-entry 3G5D, 4YBJ, 4YBK)^30,31^. Large CSPs occur predominantly around the inhibitors binding site in the active site cleft. Some residues within the C-lobe exhibit moderate CSPs suggesting that long-range allosteric effects are propagated. In the upper histogram panels, pep denotes peptide and BI denotes binding interface. Secondary structure elements β6 and β9 are absent in the structures here and in subsequent figures when the activation loop is disordered. The yellow, orange, and red histogram dashed lines here and in subsequent CSP figures indicate the magnitiude of the CSPs corresponding to the mean, 1 and 2 S.D. equal to or greater than the mean respectively

Comparison of spectra for the apo and ligand-bound states (Supplementary Fig. 2), shows that the ligands induce CSPs and IPs that are indicative of conformational and dynamic changes. In all ligand-bound states, large CSPs occur predominantly around the active site where the ligands bind (Fig. 3), suggesting local conformational changes. The identity and magnitude of the CSPs are similar for all ligands in residues spanning from the amino-terminus up to helix-αD (residues 251–350). This is likely because the ligands share a common chemical scaffold that adopts similar binding modes. However, differences occur around the binding site including β7 and β8. Here the magnitude of CSPs are largest in the dasatinib-bound state followed by DAS-DFO2 and DAS-CHO2 (Fig. 3). Although fewer long-range CSPs occur in the C-lobe, their number and magnitude differ between the ligand-bound states. For example, different patterns of CSPs occur in helices-αEF, αF, and αG. These long-range CSP differences are likely attributed to the specific effects of each conformation-selective ligand.

### Dasatinib and DAS-DFGO2 induce similar peak sharpening

IPs calculated as the ratio of normalized intensities for two states (e.g., *I*_ligand_/*I*_apo_) report on dynamic changes of the backbone amides if the oligomerisation state of the protein does not change (See Supplementary Methods). We determined that SrcKD behaves like a monomer under all experimental conditions tested here (Supplementary Fig. 3; Supplementary Table 2). IPs with a ratio smaller than 1 (broadened) suggest an increase in conformational exchange (slow internal motions) relative to fast internal motions, whereas IPs with a ratio larger than 1 (sharpened) suggest the opposite. IPs with a ratio of 1 suggests no apparent change in conformational exchange relative to fast internal motions.

**Fig. 4:**
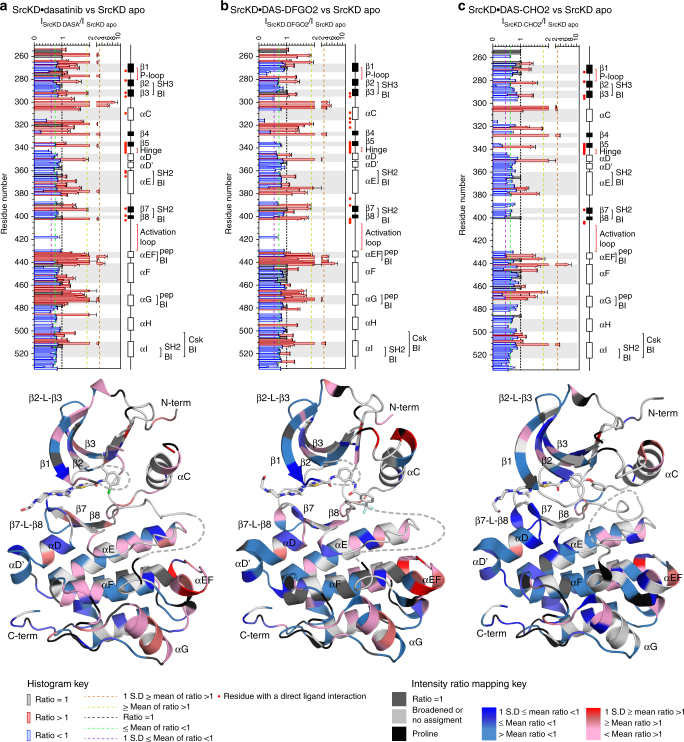
Binding of conformation-selective ligands to apo SrcKD induces short and long-range dynamic changes. Backbone amide resonance intensity ratios of SrcKD ligand-bound states: **a** dasatinib, **b** DAS-DFGO2, **c** DAS-CHO2, relative to apo SrcKD were analyzed by histograms and by structure mapping (PDB-entry 3G5D, 4YBJ, 4YBK)^30,31^ to infer dynamic changes. Intensity ratios >1 (sharpening) suggest increased fast internal motions relative to conformational exchange, intensity ratios <1 (broadening) suggest the opposite. Long-range sharpening occurs in the peptide binding interface upon dasatinib and DAS-DFGO2 binding but to a less extent for DAS-CHO2. In all states, broadening occurs in the SH3, SH2, Csk-binding interfaces but is most pronounced in the DAS-CHO2 bound state. Intensity ratios for the ligand-bound state to apo the state were determined only for resonances assigned and resolved in both states. IP error bars here and in subsequent IP figures are based on the S.D. of the noise level in the NMR spectra

We therefore compared the intensity ratios of each ligand-stabilized conformational state to identify their dynamic differences and similarities. We find that dasatinib and DAS-DFGO2 cause more sharpening than DAS-CHO2 (Fig. 4). Although some regions sharpen with all ligands, the overall pattern and extent of resonance sharpening are similar between the dasatinib- and DAS-DFGO2-bound states but show more differences when compared to the DAS-CHO2-bound state. Sharpening around the ligand binding site (Fig. 4) suggests ligand-mediated rigidification that decreases conformational exchange. Sharpening outside of the binding site may suggest redistributed fast internal motions to increase entropy upon binding. Regions around helices-αEF, αG, and αF-αG loop, undergo extensive sharpening in dasatinib and DAS-DFGO2 bound states but to a lesser extent with DAS-CHO2 (Fig. 4). Interestingly, helices-αEF and αG constitute part of the substrate peptide-binding site. Because they undergo dynamic changes, this suggests that the ATP- (where the ligands bind) and substrate peptide-binding sites are coupled, which is consistent with our previous cooperativity results in Src^19^. Another difference between the states is the extensive sharpening of the P-loop upon dasatinib binding compared to the other ligands (Fig. 4).

### Ligand binding reduces conformational exchange in the hinge

In addition to the resonance sharpening described above, all states show varying extents of resonance broadening and complete broadening due to conformational exchange on an intermediate to slow, and intermediate NMR timescale respectively^32^. Intermediate exchange broadening occurs in key regulatory elements of the apo state including: helix-αC, the hinge region and activation loop (Supplementary Fig. 4). Thus intensity ratios could not be calculated for some of these elements in the ligand-bound states. Upon dasatinib and DAS-DFGO2-binding, resonances in the hinge of the apo state become observable. This indicates that binding rigidifies the hinge, and reduces intermediate exchange broadening. In contrast, upon DAS-CHO2 binding, these hinge resonances remain intermediate exchange broadened which might be due to it binding the hinge slightly differently compared to dasatinib and DAS-DFGO2 (Supplementary Fig. 5).

### DAS-CHO2 induces long-range peak broadening

Some regions of SrcKD exhibit resonance broadening in all ligand-bound states (Fig. 4) including regulatory sites such as the SH3-binding β2-β3 loop, the SH2-binding β7-β8 loop, helices-αE, αI, and the carboxy-terminal tail. The latter two elements also overlap with the binding site of the negative Src regulator, the C-terminal Src kinase, Csk, which promotes Tyr527 phosphorylation. The overall extent and magnitude of broadening is greatest in the DAS-CHO2-bound state. Two regions in this state stand out because they show a stretch of homogenous resonance broadening and affect the SH2 and Csk-binding interface: (i) helix-αE, (ii) helix-αI to the carboxy-terminal tail. The dasatinib-bound state shows heterogeneous broadening and sharpening in region (i), but homogenous broadening in region (ii), whereas the DAS-DFGO2-bound state shows heterogeneous broadening and sharpening in both regions. The P-loop, also shows dynamic differences between the ligand-stabilized inactive and active conformations: In the DAS-DFGO2- and DAS-CHO2-bound states the central region of this element shows predominantly resonance broadening, and complete broadening due to intermediate exchange. However, when dasatinib is bound, the same region shows mostly sharpened resonances with fewer broadened. Interestingly, helix-αF which is coupled to the hydrophobic spines^33^, the allosteric network^19^, and forms a small part of the Csk-binding interface, also shows different dynamic changes in response to binding each of the ligands. Overall, DAS-CHO2 induces more extensive broadening and complete broadening across helix-αF than dasatinib and DAS-DFGO2. The above examples indicate that some of the long-range dynamic changes are ligand specific, suggesting that they are signatures of the specific ligand-stabilized conformation.

### Activation loop autophosphorylation causes widespread CSPs

**Fig. 5:**
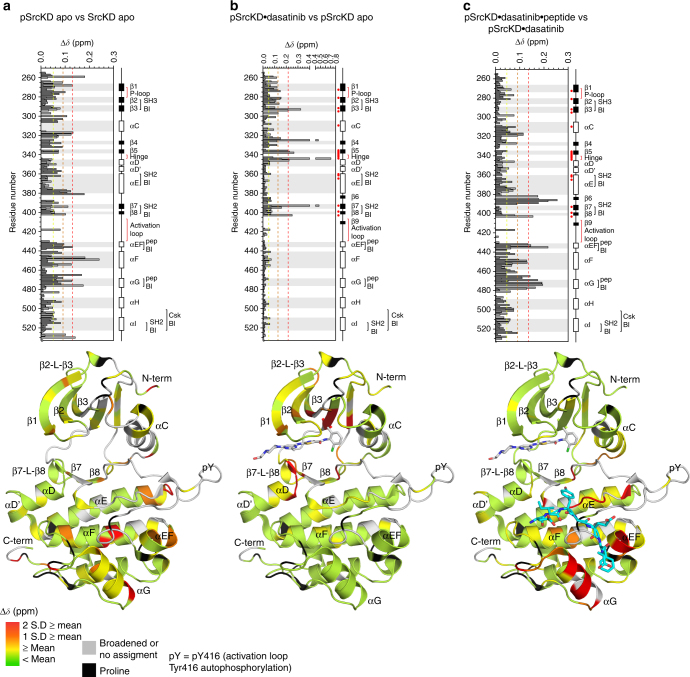
Signals that promote the active kinase conformation induce distinct CSPs at long-range sites. The CSPs induced in SrcKD by activation state stabilizing signals: **a** activation loop autophosphorylation, **b** plus dasatinib binding, **c** plus substrate peptide binding, were analyzed by histograms and structure mapping to investigate their allosteric effects. Activation loop autophosphorylation induces long-range CSPs in SrcKD affecting regulatory sites including for example helix-αF. Dasatinib binding induces CSPs predominantly around the ligand binding site with few long-range CSPs propagating to helices-αE, αF. Substrate peptide-binding induces CSPs predominantly around the substrate peptide-binding interface with long-range CSPs occurring in helices-αC, αE, and the carboxy-terminal tail. The structure was generated using PDB 1YI6, and dasatinib from PDB 3G5D, which was superimposed into the active site by structural alignment. The substrate peptide (backbone shown in cyan) was computationally docked in

To explore the conformational and dynamic changes of the active conformation and to identify potential long-range perturbations, we monitored CSPs upon activation loop autophosphorylation at Tyr416 (to yield pSrcKD). The spectrum of pSrcKD exhibits widespread CSPs relative to apo SrcKD (Supplementary Fig. 6, 7). Upon activation loop autophosphorylation, several resonances split (Supplementary Table 3), a phenomenon that suggests slow conformational exchange. We verified by mass spectrometry that SrcKD was homogenously single phosphorylated and Tyr416 was more than 90% completely phosphorylated (Supplementary Fig. 8). Mapping of the CSPs indicated that they occur throughout the N- and C-lobes, suggesting short and long-range perturbations (Fig. 5). For example, short-range CSPs occur in helix-αC, which directly interacts with the activation loop^34^. Long-range CSPs occur in regulatory sites that include: β2-β3, helices-αEF, αF, αG, and the carboxy-terminal tail. The largest CSPs occur within the center of helix-αF, which includes Gly449 despite being located ~22 Å away from the site of autophosphorylation (Fig. 5). This indicates strong long-range communication between the center of helix-αF and the activation loop. In addition, Gly449 undergoes resonance splitting upon activation loop autophosphorylation suggesting two state slow conformational exchange (Supplementary Fig. 7). Because Gly449 is located in the regulatory helix-αF and found to be highly conserved (Supplementary Fig. 9), we expect these structural and dynamic changes upon activation loop autophosphorylation to be functionally relevant.

### Dasatinib binding to pSrcKD induces few additional CSPs

Next, we investigated how binding of dasatinib to pSrcKD (to yield pSrcKD•dasatinib) affected its backbone conformation and dynamics. The spectra and CSP mapping pattern observed for this state is different from that observed upon activation loop autophosphorylation of SrcKD, and dasatinib binding to SrcKD (Figs. 3, 5; Supplementary Figs. 2, 6). Fewer CSPs occur in pSrcKD•dasatinib and they predominantly originate within the N-lobe. Long-range C-lobe CSPs appear in helices-αE, αF (Fig. 5). In the N-lobe, the amino-terminal end of helix-αC shows minimal CSPs, indicating that these residues remain unaffected by dasatinib binding to pSrcKD, whereas activation loop autophosphorylation can perturb them (Fig. 5). In contrast, the β2-β3 and β7–β8 loop regions (part of the SH3 and SH2-binding interface respectively) which were unperturbed in pSrcKD are perturbed in SrcKD•dasatinib and pSrcKD•dasatinib (Figs. 3, 5). Both helices-αEF and αG in the C-lobe show CSPs in pSrcKD and SrcKD•dasatinib, but in pSrcKD•dasatinib, no additional CSPs relative to pSrcKD occur. This suggests that helices-αEF, αG in pSrcKD and pSrcKD•dasatinib are in a similar conformation. Upon dasatinib binding to pSrcKD many resonances also retain the resonance splitting phenomenon (Supplementary Figs. 6, 7; Supplementary Table 3), suggesting that slow conformational exchange in these residues remain mostly unperturbed. Interestingly, Gly449 undergoes additional CSPs upon dasatinib binding to pSrcKD (Supplementary Fig. 7), suggesting further structural changes are induced in the center of helix-αF.

### Dasatinib and peptide binding dampen distal CSPs in pSrcKD

Next, we tested whether substrate peptide binding to pSrcKD•dasatinib (to yield pSrcKD•dasatinib•peptide) could induce further perturbations including long-range effects from the C-lobe substrate peptide-binding site. We showed previously that Src binds ATP and substrate peptide with negative cooperativity^19^. Because of coupling between the ATP- and substrate peptide-binding sites, we expected to observe long-range CSPs between them. Spectral comparison of pSrcKD•dasatinib•peptide and pSrcKD•dasatinib states indicated that widespread CSPs occur upon substrate peptide binding (Supplementary Fig. 6). We found that substrate peptide binding induces additional local CSPs mostly around the substrate peptide-binding site (Fig. 5). In the C-lobe, long-range CSPs occur in helices-αD, αE, αI, the carboxy-terminal tail, and β6. However, fewer long-range CSPs occur in the N-lobe in comparison to the effect of activation loop autophosphorylation of SrcKD. This may be because the pSrcKD•dasatinib state is rigidified by dasatinib, a high affinity ligand which could dampen the propagation of CSPs induced by substrate peptide binding. Most of the split resonances persist after substrate peptide binding, but some converge to one resonance or broaden out into intermediate exchange, suggesting a shift in their dynamic behavior.

### pSrcKD shows peak sharpening similar to SrcKD•dasatinib

**Fig. 6:**
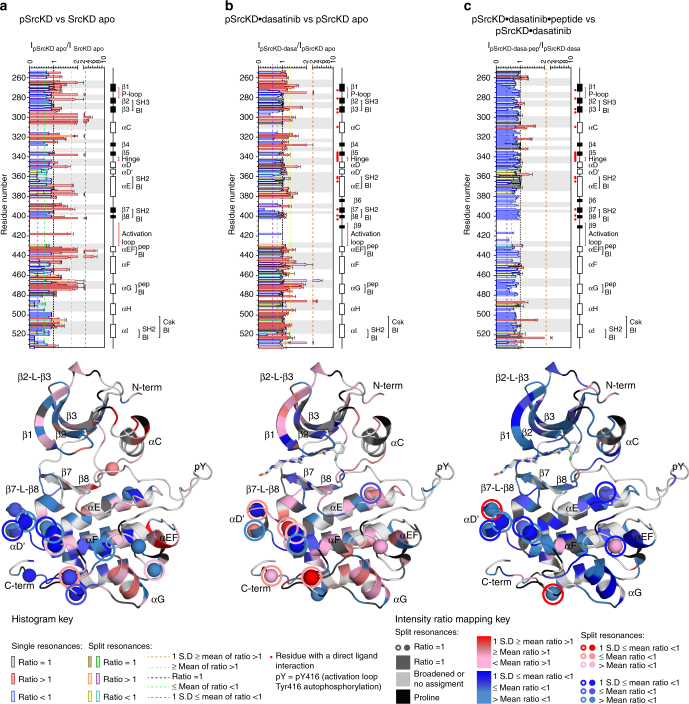
Signals promoting the active conformation induce long-range dynamic changes. Backbone amide resonance intensity ratios of active SrcKD states formed upon: **a** activation loop autophosphorylation, (**b**) plus dasatinib, binding **c** plus substrate peptide binding, were analyzed by histograms and by structure mapping (PDB-entry 3G5D, 4YBJ, 4YBK)^30,31^ to infer dynamic changes and interpreted as in Fig. 3. Spheres and circles denote split resonances (Supplementary Table 3). Activation loop autophosphorylation causes long-range sharpening and broadening affecting regulatory sites. Dasatinib induces additional sharpening to activation loop autophosphorylation in some regions, while in other regions it sharpens regions previously broadened by activation loop autophosphorylation. Peptide binding induces overall broadening, reverting the sharpening effects induced by dasatinib. Some intensity ratios for the second split resonance (circle) were omitted because of overlap or for clarity because its intensity ratio is similar to the first resonance in terms of magnitude as shown in the key

Stabilizing SrcKD in specific conformational states through the binding of conformational-selective ligands induces long-range dynamic changes (Fig. 4). Therefore we expected that activation loop autophosphorylation which stabilizes the active kinase conformation would have similar effects. In fact, we find that activation loop autophosphorylation of apo SrcKD, and dasatinib binding to SrcKD, both show resonance sharpening in similar regions (Figs. 4, 6). The extensive sharpening across helices-αEF, αG, in pSrcKD suggests that activation loop autophosphorylation modulates the dynamics of these peptide binding elements in a similar manner to dasatinib binding to apo SrcKD. These similarities may indicate a common active conformation that both inputs stabilize. Resonances spanning from β3 to β5 which includes helix-αC, were previously completely broadened out into intermediate exchange in apo SrcKD but sharpen upon activation loop autophosphorylation. This suggests that activation loop autophosphorylation mediated rigidification reduces conformational exchange in this region (Supplementary Fig. 10). In addition, activation loop autophosphorylation of apo SrcKD results in an overall increase in resonance broadening compared to the ligand-bound states for (i) helix-αD’ to the amino-terminal end of helix-αE, (ii) helix-αF, (iii) αG-αH loop, to helix- αI (iv), and the carboxy-terminal tail (Figs. 4, 6). As some of these regions corresponds to regulatory sites such as helix-αF, and the SH2, and Csk-binding interfaces, this suggests that activation loop autophosphorylation modulates these regulatory sites differently from the ligands. Finally, autophosphorylation causes resonance splitting in a region of the C-lobe that includes elements near the SH2 and Csk-binding interfaces, which further suggests that activation loop autophosphorylation might modulate these regulatory sites through long-range dynamic changes.

### Dasatinib and peptide cause opposing dynamic changes

Upon dasatinib binding to pSrcKD (pSrcKD•dasatinib), resonance sharpening and broadening generally occurs in similar regions to those seen in the pSrcKD state (Fig. 6; Supplementary Fig. 10). This suggests that dasatinib induces dynamic changes in pSrcKD•dasatinib that are similar to those induced by activation loop autophosphorylation in pSrcKD, but some regions also suggest opposing dynamic changes through contrasting sharpening/broadening effects. C-lobe elements which undergo extensive resonance sharpening/reduced broadening include helices-αEF, αF, αG, and αH to the carboxy-terminal tail. Notably the magnitude of sharpening in helix-αEF is much smaller than in helix-αG (Fig. 6). This is different from activation loop autophosphorylation of apo SrcKD, and dasatinib binding to apo SrcKD (Figs. 4, 6), where both helices-αEF, αG undergo significant sharpening. Differences in the dynamic changes are also apparent in helix-αF and from helix-αH to the carboxy-terminal tail in pSrcKD•dasatinib. These elements show sharpening/reduced broadening effects upon dasatinib binding to pSrcKD, which contrast with their broadening effects upon activation loop autophosphorylation of apo SrcKD. This suggests that the dasatinib induced sharpening dominates the autophosphorylation induced broadening. Thus the combination of autophosphorylation and dasatinib binding, compared to autophosphorylation alone, leads to distinct modulation of the dynamics for these non-regulatory and regulatory sites. Of the SH2-binding elements in pSrcKD•dasatinib only the carboxy-terminal tail is affected by pronounced sharpening/reduced broadening upon dasatinib binding. This implies another dynamic behavior that is specific to the pSrcKD•dasatinib state.

Upon substrate peptide binding to pSrcKD•dasatinib, resonance broadening occurs throughout the N- and C-lobes suggesting long-range effects not observed through CSPs (Figs. 5, 6). Notably, helix-αF, and the region from helix-αH to the carboxy-terminal tail broaden further. These regions previously showed sharpening/reduced broadening when dasatinib was bound to pSrcKD but revert to broadening upon substrate peptide binding (Fig. 6). Resonance broadening in pSrcKD•dasatinib•peptide is most pronounced around helices-αF, αG, which forms part of the peptide binding site (Fig. 6). This indicates that these local broadening effects report on a combination of conformational changes, ligand binding, and changes in solvent exposure. However, resonances that were broadened into intermediate exchange in pSrcKD•dasatinib largely remain in this dynamic state upon substrate peptide binding (Supplementary Fig. 10). This contrasts with the effects of activation loop autophosphorylation of SrcKD and dasatinib binding to pSrcKD, which can reduce intermediate exchange broadened resonances (Supplementary Fig. 10).

To summarize, we observe that activation loop autophosphorylation causes dynamic changes in apo SrcKD similar to those of dasatinib binding to SrcKD: resonance broadening occurs in similar regions but the magnitude of broadening is greater upon phosphorylation. When we combine autophosphorylation with dasatinib binding to produce pSrcKD•dasatinib, we observe that many of the same regions sharpen in pSrcKD upon autophosphorylation of apo SrcKD, and in SrcKD•dasatinib upon dasatinib binding. Importantly, regulatory sites that were broadened upon autophosphorylation of SrcKD, sharpen upon dasatinib binding to pSrcKD. In contrast, substrate peptide binding to pSrcKD•dasatinib causes overall resonance broadening, indicating more global dynamic changes.

### Fast internal motions dominate the pSrcKD•dasatinib state

**Fig. 7:**
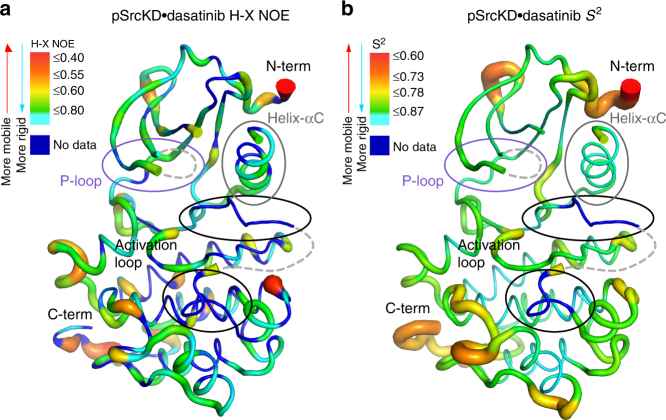
Fast backbone dynamics are present at distal sites of pSrcKD•dasatinib. **a** The H-X NOE ratio which indicates motions on the pico- to nanosecond timescale are mapped to the structure of SrcKD•dasatinib (PDB: 3G5D). **b** *S* ^2^ order parameters predicted from NMR chemical shifts report on the amplitude of displacement of the backbone amide bond vector from its mean position on the pico- to nanosecond dynamics timescale (fast internal motions). The H-X NOE ratio and *S* ^2^ values are indicative of flexible and rigid backbone regions. Both show similar regions of flexibility and rigidity. The thickness of the backbone and the corresponding color shown in the key reflects the extent of flexibility/rigidity. Here dasatinib is omitted from the structure for clarity

We were intrigued by the dynamics of the pSrcKD•dasatinib state because split resonances indicated slow conformational exchange whereas resonance sharpening indicated rigidification and increased fast internal motions. Therefore, we decided to directly measure the dynamics of the pSrcKD•dasatinib backbone on the pico- nanosecond timescale through *R*_*1*_, *R*_*2*_ and heteronuclear NOE relaxation experiments (Supplementary Fig. 11). Backbone relaxation measurements can distinguish rigid residues with dynamics defined by the global tumbling rate of the molecule from those with added fast or slow internal motions. On the basis of the NMR relaxation data, some parts of the protein backbone, including secondary structure elements and those in contact with dasatinib are relatively rigid. These residues tumble with the global tumbling rate of the protein of 20.65 ns which is similar to the predicted value of 21.5 ns (Supplementary Table 4). In addition to these rigid residues, our data indicate the existence of residues with fast internal motions in the pSrcKD•dasatinib state and is consistent with our IP analysis that suggested fast internal motion contributions (Fig. 6). Interestingly, residues indicating fast internal motions include those from the β2–β3 loop and the carboxy-terminus (Supplementary Fig. 11). Furthermore, heteronuclear NOE ratios identify residues undergoing fast internal motions in secondary structure elements and loops throughout the backbone, both near and distant from the dasatinib binding site and autophosphorylation site at Tyr416 (Fig. 7, Supplementary Fig. 11). The extent of rigidity and flexibility can also be inferred from chemical shift derived order parameters *S* ^2^, which describes the amplitude of displacement of an amide bond vector from its average position for fast internal motions^35^. Mapping of the *S* ^2^ values for the pSrcKD•dasatinib state to the SrcKD structure (Fig. 7) shows regions of rigidity and fast internal motions that are consistent with those observed in the heteronuclear NOE measurements. The backbone relaxation experiments also identify residues that might undergo conformational exchange (Supplementary Fig. 11). We find that such residues in pSrcKD•dasatinib include those in the SH2-binding elements: helix-αE and the β7–β8 loop.

### Gly449 mediates full activation of Src3D

Because Gly449 neighbors the allosteric network and undergoes a large long-range CSP concomitant with resonance splitting upon activation loop autophosphorylation, we postulated that it might be implicated in mediating maximal Src activity. We found that the G449A mutant has no effect on the kinase activity of SrcKD and pSrcKD (Supplementary Fig. 12). However, activation loop autophosphorylation of a Src construct containing the regulatory SH3 and SH2 domains and SrcKD (Src3D) increased the specific activity by 31% to the level of SrcKD, whereas the activity of Src3D_G449A increased by only ~8%. This indicated that G449 is necessary for full activation of Src3D by activation loop autophosphorylation. As Gly449 is near the peptide binding site, which is coupled to the ATP-binding site, it is possible that G449A affects kinase activity by affecting substrate affinity. We therefore determined the *K*_m_ for ATP and peptide for wild type and mutant SrcKD and Src3D and found that G449A behaves like wild type (Supplementary Fig. 13; Supplementary Table 5). Interestingly, spectral comparison of wild type and mutant SrcKD•dasatinib states in the absence and presence of autophosphorylation shows G449A dependent differences in CSPs and resonance splitting (Supplementary Fig. 14; Supplementary Table 3), suggesting that G449A has altered the perturbation effects of activation loop autophosphorylation and dasatinib binding. Analysis of these perturbations indicates that G449A induces CSPs predominantly around helix-αF in both unphosphorylated and autophosphorylated states (Supplementary Fig. 15), and extensive broadening in the former state but substantial sharpening in regions of the latter state such as the carboxy-terminal tail (Supplementary Fig. 16). Autophosphorylation-dependent CSPs and IPs in the presence of G499A were also analyzed and compared to wild type. Here the magnitude of the CSPs around helix-αF in the mutant are smaller than wild type (Supplementary Fig. 15), suggesting attenuation of crosstalk between helix-αF and the activation loop which is consistent with our observed activation defect in Src3D_G449A. The IPs due to autophosphorylation in the mutant state indicate that extensive sharpening occurs throughout (Supplementary Fig. 16). In certain regions such as the SH2, Csk-binding interfaces, this contrasts with the wild type state where broadening occurs instead (Supplementary Fig. 16).

Because only pSrc3D_G449A shows an activation defect in biochemical assays (Supplementary Fig. 12), the structural and dynamic changes caused by G449A likely require the SH3–SH2 domains in the Src3D construct to prevent kinase activation. These results suggest that the compact structure Gly449 and its wide range of accessible dihedral angles allows structural and dynamic changes in helix-αF that are necessary for communicating regulation of Src3D activity by activation loop autophosphorylation.

### G449A stabilizes the assembled over the disassembled state

Because G449A prevents full activation only in Src3D (Supplementary Fig. 12), we were further intrigued about its mechanism of action and whether changes in protein stability occurred in addition to the above structural and dynamic changes. We used Foldx^36–38^, an empirical method for calculating the effect of amino acid substitution upon protein stability. G449A destabilizes the disassembled active conformation of Src3D by ~1 kcal mol^−1^ more than the assembled inactive conformation due to an increase in Van der Waal clashes (Supplementary Figs. 17, 18). In contrast, G449A destabilized all SrcKD conformations to a similar extent. Our data suggest that interactions between the regulatory domains and kinase domain in the assembled inactive conformation of Src3D induces a C-lobe conformation that is less tightly packed around helix-αF and is therefore more tolerant of G449A compared to the disassembled active conformation.

### The SH3–SH2 domains interact with SrcKD in Src3D•dasatinib

Full length Src features regulatory SH3–SH2 domains which interact with SrcKD and therefore must impact its conformation, dynamics, and activity^4^. Moreover, we have shown that the activity of Src3D increases in response to activation loop autophosphorylation, and is mediated by Gly449, suggesting long-range communication between the activation loop and regulatory domains. However, studying large dynamic protein kinases by NMR is challenging and often requires labeling strategies to reduce spectral crowding as demonstrated by similar kinases: Abl and Csk^39,40^. To begin probing Src3D, we used sortase-mediated protein ligation to fuse the SH3–SH2 and SrcKD domains^41^. Selective ^15^N-labeling of either the regulatory or catalytic domains allowed us to reduce spectral crowding (Supplementary Fig. 19) whilst retaining activity comparable to wild type (Supplementary Fig. 20). The two Src3D fusion constructs created were: (i) ^15N^SH3-^15N^SH2-SrcKD, (ii) SH3-SH2-^15N^SrcKD.

**Fig. 8:**
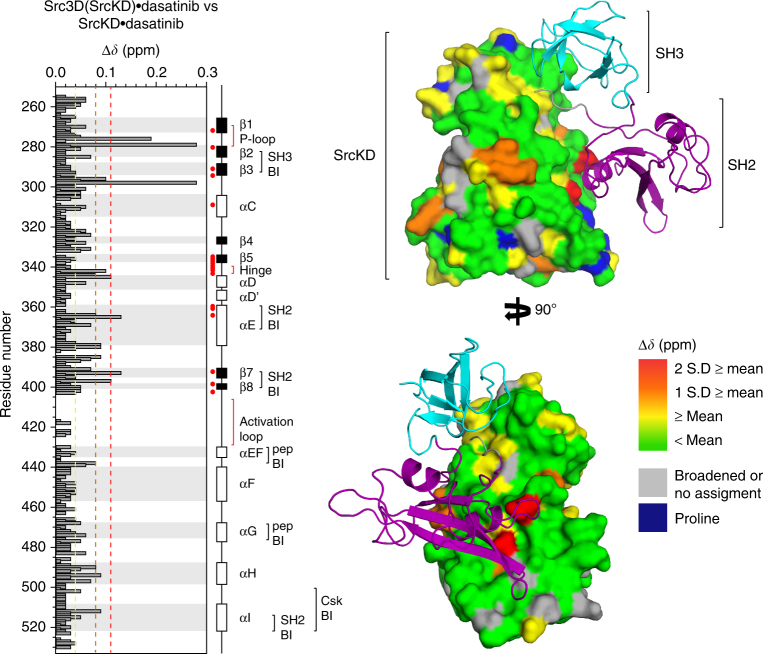
The SH3–SH2 domains interact with the dasatinib stabilized kinase domain of a Src3D fusion construct. Histogram and structure mapping analysis of CSPs between isolated SrcKD•dasatinib and SrcKD•dasatinib from Src3D fusion•dasatinib, suggest an interaction between the regulatory domains and the kinase domain in the context of Src3D fusion•dasatinib. The CSPs were mapped to the structure of SrcKD•dasatinib (PDB: 3G5D) with the regulatory SH3–SH2 domains from near full length Src (PDB: 2SRC PDB) superimposed, indicating that they interact with the SH3 and SH2-binding interfaces. As shown in the histogram the SH3-binding interface comprises of the SH2-SrcKD linker, β2-β3 loop, the SH2-binding site comprises of: helix-E, β7-β8 loop, helix-αI, and the carboxy-terminal tail

**Fig. 9:**
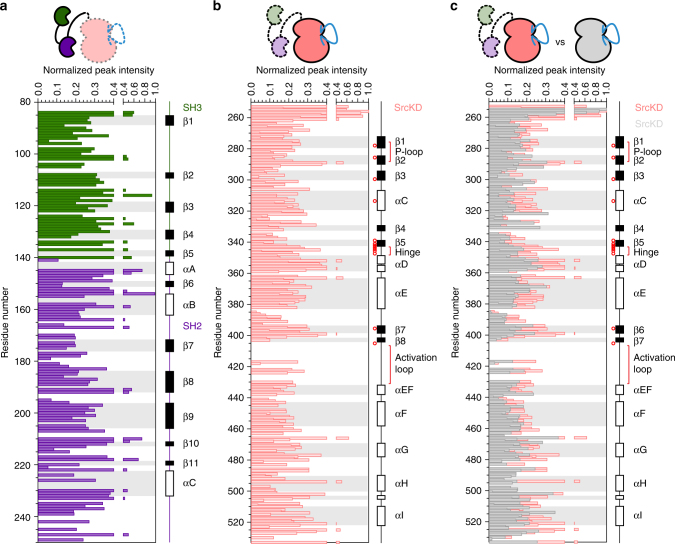
Resonance intensities in Src3D resemble those of SrcKD indicating conservation of global backbone dynamics. Histograms of normalized backbone amide resonance intensities are shown for Src3D fusion•dasatinib constructs and SrcKD•dasatinib. **a** Heterogeneous resonance intensities span across the SH3 (green), SH2 (purple) domains in Src3D fusion•dasatinib, and **b** SrcKD (pink) of Src3D fusion•dasatinib. **c** A comparison of the resonances intensities for SrcKD from Src3D fusion•dasatinib and isolated SrcKD•dasatinib indicates that the both constructs follow a similar trend of heterogeneous intensities overall, but also show local differences. The Src3D fusion constructs ^15N^SH3-^15N^SH2-SrcKD and SH3-SH2-^15N^SrcKD, and the isolated ^15N^SrcKD, are shown schematically above their corresponding histograms. Domains which are ^15^N selectively labeled and unlabeled domains are indicated by solid lines and colors, and dashed lines and faded colors respectively

To investigate the effects of the SH3–SH2 domains on SrcKD•dasatinib in the context of Src3D•dasatinib, we analyzed CSPs between a Src3D fusion•dasatinib construct (SH3-SH2-^15N^SrcKD•dasatinib) and SrcKD•dasatinib. CSP mapping suggests that the SH3–SH2 domains interact with the SH3–SH2 interface on SrcKD transiently because few CSPs exhibited large magnitudes (Fig. 8). Some of the SrcKD regions that exhibit such changes are strand β2, β7, helices-αC, αE, and αI. Interestingly, some of the CSPs also overlap with the dasatinib binding site. Because the carboxy-terminal tail of the Src3D fusion construct is not-phosphorylated, the transient nature of the SH3–SH2 and SrcKD interaction suggests that Src3D fusion•dasatinib interconverts between disassembled and assembled states. Furthermore, this transient inhibitory interaction is consistent with the lower basal activity of Src3D (Supplementary Fig. 12), indicating that the SH3–SH2 domains, even in the absence of carboxy-terminal tail phosphorylation, can interact with SrcKD in an autoinhibitory manner and reduce its activity. In addition the normalized resonance intensities of the SH3–SH2 domains, and SrcKD in Src3D fusion•dasatinib are heterogeneous across their secondary structure elements and loops (Fig. 9). This suggests that these domains are dynamic and not completely rigid. The CSPs between the SrcKD of Src3D fusion•dasatinib and isolated SrcKD•dasatinib are consistent with MD simulation data on Hck, a related Src-family kinase, in which the SH3–SH2 domains did not completely disengage from the kinase domain despite its C-terminal tail being unphosphorylated^42^. In summary, by using our Src3D fusion constructs we are able to monitor the CSPs that are induced by the SH3–SH2 domains in SrcKD. Because these CSPs localize around the SH3–SH2-binding interface our results indicate that the regulatory and catalytic domains interact in the Src3D fusion•dasatinib state.

## Discussion

We have shown that long-range CSPs and IPs can propagate throughout the backbone of SrcKD upon ligand binding, activation loop autophosphorylation and substrate peptide binding. Our results indicate that long-range communication is predominantly mediated by dynamic changes rather than conformational changes, a phenomenon that has been reported in other studies^25^. Moreover, it further supports the allosteric network model which spans SrcKD. For example, dasatinib binding to SrcKD perturbs the dynamics of the substrate peptide-binding interface. This suggests that dynamic changes modulate the negative cooperativity of substrate binding between the sites which is something we proposed recently based on MD simulations and biochemical experiments^19^. Some of the dynamic changes in the regulatory SH3-, SH2-, and Csk-binding interfaces were specific to the conformation-selective ligand. This implies that the conformation of the active site affects binding of regulatory domains and Csk via long-range dynamic changes. For example, stabilization of the “DFG-Asp-in/αC-out” inactive conformation by DAS-CHO2 binding caused broadening in helix-αI and the carboxy-terminal tail, suggesting that it modulates the SH2 and Csk-binding interface. Interestingly, it has been shown in Src and the closely related Hck kinase, that conformation-selective ligands specifically modulate the accessibility of the SH3, SH2 domains, interactions with Csk, and PTP1B phosphatase^43^. Here, “DFG-Asp-in/αC-out” inhibitors were found to reduce the accessibility of SH3–SH2 domains for their respective immobilized binding peptides and enhance their autoinhibitory interactions with the kinase domain thus stabilizing the assembled inactive state. Furthermore, this contrasted with the effects of “DFG-Asp-out/αC-in” inhibitors. Therefore, we propose that the resonance broadening in helix-αI and the carboxy-terminal tail indicate dynamic changes that affects its interactions with inhibitory factors (SH2 and Csk) and assembly of the inactive conformation. As allosteric communication is bidirectional these results also suggest that dynamic changes in the C-terminal tail can be communicated to the active site to favor the “DFG-Asp-in/αC-out” inactive conformation.

Similar long-range communication has been observed previously. For example, hydrogen-deuterium exchange studies on Src3D have reported long-range effects upon imatinib binding on the timescale of seconds and identified perturbations through the hydrophobic spine to the SH3–SH2 domains^44^. A difference in these studies apart from the timescale is that imatinib binds with micromolar affinity to Src kinase in the DFG-Asp-out conformation, whereas DAS-DFGO2 used here binds with nanomolar affinity to Src kinase. ATP-competitive inhibitors have also been shown to modulate long-range regulatory interactions in MAPKs with divergent outcomes^23^: even though these inhibitors effectively reduced enzymatic kinase activity, some inhibitors activated MAPK signaling pathways, whereas some inhibitors inhibited MAPK signaling pathways. This indicates that the kinase conformation itself exhibits some signaling activity (e.g., through scaffolding, etc.) independent of enzymatic activity. It would be interesting for future investigations to see if the conformational-selective inhibitors used here cause different signaling outcomes for Src and if these differences relate to the conformational and dynamic changes that the inhibitors induce in their target kinases.

We wanted to understand how the combination of different activating inputs affects kinase dynamics. We were surprised to find these factors caused similar dynamic responses in some regions but opposite responses in other regions of the kinase. Dasatinib binding or activation loop phosphorylation rigidified similar regions which suggests that these inputs stabilized similar active conformations. The combination of activation loop phosphorylation with dasatinib binding rigidified these regions further. In contrast to the overall sharpening effects of activation loop phosphorylation and dasatinib binding, substrate peptide binding to pSrcKD•dasatinib induces extensive broadening. Interestingly, this contrasting dynamic effect of ligand binding to the ATP-binding site and to the substrate peptide-binding site points at a dynamic origin of the observed anti-cooperative binding of substrate peptide and ATP^19^. Interestingly, the Ser/Thr kinase, PKA, exhibits the opposite behavior: PKA binds ATP and substrate peptide with positive cooperativity and exhibits an overall increase in fast internal motions^45^. It is therefore tempting to speculate that the correlation between cooperativity of substrate binding and effect on protein dynamics points to a dynamic origin for the allosteric communication between the substrate-binding sites.

Although the binding affinity and binding kinetics of dasatinib to SrcKD are independent of activation loop phosphorylation, binding of imatinib to Abl kinase highly depends on activation loop phosphorylation^30,46–48^. We observe resonance sharpening within the P-loop upon activation loop autophosphorylation of SrcKD, indicating crosstalk between these two regulatory elements. Interestingly, dasatinib binding to unphosphorylated Src also induces sharpening in the P-loop. This is surprising since dasatinib does not directly interact with the P-loop^31,49,50^. The observed resonance sharpening therefore suggests indirect effects as the cause for the dynamic changes. Binding of dasatinib to pSrcKD causes slight broadening in the P-loop. This suggests that dasatinib weakly modulates crosstalk between the P-loop and activation loop differentially in unphosphorylated and phosphorylated states. Interestingly, P-loop mutations are frequently reported to disrupt interactions with inhibitors, to increase the flexibility of this loop, and to alter the conformation of the activation loop^51–54^.

Overall, our results demonstrate dynamic crosstalk between the activation loop and the P-loop in SrcKD that is sensitive to activation loop phosphorylation and dasatinib binding. This implies that ligand binding affecting the P-loop causes dynamic changes at the activation loop where substrate peptides bind and vice versa. In addition, our observations provide experimental support for the model that resistance mutations in the P-loop can act by altering protein dynamics.

The redistribution of internal motions to distant elements and the transient unfolding of distal helices are mechanisms to compensate the entropic penalty of ligand-mediated rigidification^55^. Here, we cannot rule out the possibility that our observed long-range dynamic perturbations are accompanied with transient unfolding events to compensate entropic penalties upon ligand binding. Our identification of long-range perturbations in regulatory and non-regulatory sites might also yield insight into rationalizing drug resistance mutations distant from the drug binding site. These sites may correspond to mutation hotspots that rigidify entropic sinks. Resistance could also be conferred at these site by disrupting allosteric regulation of autoinhibitory signals through conformational and dynamic changes that perturb their autoinhibitory function^44^.

The highly conserved Gly449 residue is one of the long-range allosteric sites we found upon activation loop autophosphorylation. Gly449 is necessary for full activation of Src3D upon activation loop autophosphorylation. Interestingly, Gly449 occupies the center of helix-αF, an element which anchors the hydrophobic spines^33^. We found that G449A is predicted to destabilize the Src3D assembled inactive state less than the disassembled active state which would result in an increase in the inactive kinase population and an overall reduction in kinase activity, consistent with our observation. In contrast, the mutation destabilized all three conformations of SrcKD to the same extent, leaving the population of active SrcKD unaltered, again consistent with our experimental observation that the specific activity of SrcKD wild type and G449A were the same. Our results suggest that the inherent flexibility of Gly449 and its lack of a side chain is important for regulation of Src3D. We speculate that these properties allows the center of helix-αF to propagate activating signals from activation loop autophosphorylation; and to function as a sink for strains induced by the regulatory domains upon their engagement and disengagement from the kinase domain.

Next, we studied by NMR how autoinhibitory signals from the SH3–SH2 domains are integrated into the kinase domain. Our comparison of SrcKD•dasatinib and SrcKD from Src3D fusion•dasatinib suggested that the SH3–SH2 domains perturb the kinase domain even in the absence of Tyr527 phosphorylation. Because CSPs with moderate magnitude occurred near the binding sites for SH3 and SH2 domains, we believe this indicates a weak interaction between the regulatory domains and the kinase domain suggesting a partially assembled inactive-like state consistent with the findings from other studies^56,57^. On the basis of our observation that binding of dasatinib to apo SrcKD induces dynamic changes in the binding sites for the SH3 and SH2, we speculate that dasatinib could modulate the transient interactions between the regulatory domains and the kinase domain in Src3D•dasatinib. Similar crosstalk between the ATP-binding site ligand and regulatory domains has been observed for Abl kinase, whereby the ATP-competitive ligand imatinib, synergizes with an allosteric ligand to stabilize the assembled inactive state^58^.

In summary, we have investigated the effects of conformation-selective ligands and active inputs on the backbone conformation and dynamics of SrcKD. We discovered short and long-range effects that perturb regulatory and non-regulatory sites. Distinct CSPs and IPs were apparent, suggesting conformation-selective ligand and active input-specific effects. This likely reflects the distinct mechanisms by which they stabilize their respective conformations. Similar IPs were also observed between activation loop autophosphorylation and dasatinib binding, suggesting that their stabilized active conformations have common dynamic changes in SrcKD. The identification of distal sites which show dynamic changes might help to rationalize distal cancer resistance mutations if they are implicated in mediating autoinhibitory interactions or entropy compensation mechanisms (e.g., transient unfolding, fast internal motion redistribution). Our studies with Src3D fusion allows for follow up studies that probe long-range perturbations within SrcKD in the presence of the SH3–SH2 domains and appreciate how their regulatory signals are integrated with ligand binding and activation loop autophosphorylation.

## Methods

### SrcKD, Src3D, and SH3–SH2 protein sample preparation

Chicken SrcKD encompassing residues 251–533 was cloned, expressed and purified in bacteria with additional co-expression of the GroEL chaperone^59^. Briefly, Src kinase domain containing a TEV-cleavable N-terminal His_6_-tag was co-transformed into *E. coli* BL21 (DE3) cells with plasmids expressing YopH phosphatase and GroEL and Trigger factor. Mutations were introduced by site-directed mutagenesis and verified DNA sequencing. Perdeuterated ^15^N, ^13^C, SrcKD samples for triple resonance experiments were produced in 100% ^2^H_2_O M9 minimal media containing 1 g L^−1^ of ^15^NH_4_Cl and d-glucose-^13^C-^2^H_7_ as the sole nitrogen and carbon sources, respectively. Cells were grown at 37 °C to O.D_600nm_ ~0.3–0.4 then cooled to 16 °C. At O.D_600nm_ ~0.6–0.8, expression was induced with 0.5 mM IPTG and allowed to continue overnight. Deuterated ^15^N, and non-deuterated ^15^N samples were grown similarly in 80% ^2^H_2_O, or H_2_O respectively but using M9 minimal media with regular d-glucose. Phe, Val, and Tyr residue-specific ^15^N labeled samples were prepared by growing cells in H_2_O-based rich media or modified unlabeled M9 minimal media containing NH_4_Cl and d-glucose in which all amino acids added were unlabeled. These cells were also grown and induced to express protein as described for the triple (^2^H, ^15^N, ^13^C) double (^2^H, ^15^N), and single (^15^N) labled samples. Src3D encompassing residues 82–533, was expressed in BL21 DE3 cells with YopH and GroEL/Trigger Factor, and purified similarly to SrcKD. The SH3–SH2 domains comprised of residues 82–253 were cloned and expressed in BL21 DE3, and again purified under similar conditions.

Proteins were purified by Ni-NTA affinity, anion exchange and size exclusion chromatography. Purified SrcKD was autophosphorylated within the activation loop to promote the active conformation^60^ by incubating with 0.5 mM ATP, 10 mM MgCl_2_, and 2 mM Na orthovanadate. To maintain stable activation loop phosphorylation, 2 mM Na_3_VO_4_ was added to the sample post autophosphorylation. For deuterated NMR samples, the exchange of ^2^HN for ^1^HN at labile backbone amide positions was performed by incubating the sample in 20 mM Tris pH 8, 250 mM NaCl, 1 mM TCEP at 4 °C. Post exchange, the sample was concentrated by ultrafiltration and buffer exchanged into 50 mM MES pH 6.4, 300 mM NaCl, 1 mM TCEP. To reduce aggregation of protein samples at concentrations >200 µM, 50 mM Arg, Glu was added. For samples up to 200 µM, addition of 50 mM Arg, Glu was not necessary. In final NMR samples, 10% ^2^H_2_O was added for a lock signal. ATP-competitive ligands (dasatinib, DAS-DFGO2, DAS-CHO2) and the substrate peptide (AEEEIYGEFAKKK) were added in excess accordingly to saturate the ATP- and substrate peptide-binding sites and stabilize particular SrcKD states. D^6^-DMSO was maintained at 5% for NMR samples containing ligands solubilized in D^6^-DMSO.

### Src3D fusion sample preparation

Selective ^15^N-labeling of only the SH3–SH2, or SrcKD portions of full length Src (Src3D) was achieved using sortase to mediate post translational fusion of the independently expressed and purified SH3–SH2, and SrcKD domains, which created a Src3D fusion construct. To facilitate the fusion, mutations were engineered into SH3–SH2 and SrcKD that enabled their recognition and ligation as substrate and target proteins by sortase. The SH3–SH2 carboxy-terminal residues KPQTQG (249–254), were mutated to LPQTGG to engineer a LPXTGG sortase substrate protein recognition motif. SrcKD amino-terminal residues HMQTQ, which follows after the TEV cleavage site Gly residue, were replaced with G to yield the GG motif post TEV cleavage which enabled it to become the target protein. These mutations also preserved the length of the endogenous linker between the SH2 and SrcKD and introduced only two amino acid substitutions: wild type—KPQTQG to mutant fusion—LPQTGG. Ligation between the SH3–SH2 and SrcKD was performed by incubating the proteins with sortase at final concentrations of 50 µM, overnight at 30 °C in 1× reaction buffer: 50 mM Tris pH 8, 150 mM NaCl, 10 mM CaCl_2_. Post ligation, the Src3D fusion was purified from sortase and unligated SH3–SH2 and SrcKD by size exclusion in 20 mM Tris pH 8, 250 mM NaCl, 1 mM TCEP.

### Kinase activity assays

A continuous spectrophotometric coupled assay^61^ was used with 250–500 µM ATP, 300–900 µM Src optimal substrate peptide (AEEEIYGEFAKKK), in 50 mM Tris pH 7.5, 10 mM MgCl_2_ at 30 °C.

### CSP and IP experiments

All spectra measuring CSPs and IPs were collected on a Bruker Avance III HD spectrometer operating at a ^1^H frequency of 850 MHZ equipped with a cryogenic probe using ^1^H-^15^N TROSY-HSQC experiments^62–67^ at 30 °C. Here NMR samples contained ~180 µM protein in 50 mM MES pH 6.4, 300 mM NaCl, 1 mM TCEP, 10% ^2^H_2_O. An additional 2 mM Na_3_VO_4_ was added for autophosphorylated samples. Inhibitors were added in 2-fold excess, with 5% D6-DMSO, whilst substrate peptide prepared in 50 mM MES pH 6.4 was added in 10-fold excess. CSP measurements were weighted according to the formula ((Δ*δ*_H_)^2^ + (0.2*Δ*δ*_N_)^2^)^0.5^. For the IP measurements, resonance intensities were normalized to account for sample to sample variability. Intensities were normalized against the average intensity of the resolved amino-terminal residues 255–256. These were selected because in all SrcKD states they typically exhibited the highest intensities, and were far enough away from the ligand binding and activation loop autophosphorylation sites that they showed minimal CSPs (See Supplementary Methods for further IP details). For the normalized resonance intensity analysis of Src3D fusion, the resonances were normalized against residue 256. Errors in the intensity ratio analysis were calculated through error propagation using the root mean square noise of the spectra.

### Backbone relaxation experiments

All backbone dynamics ^15^N *T*_1_, *T*_2_, and (^1^H)-^15^N heteronuclear NOE relaxation experiments were acquired at 30 °C on a Bruker Avance III HD spectrometer operating at a ^1^H frequency of 850 MHz equipped with a cryogenic probe using pSrcKD•dasatinib ^2^H-^15^N samples prepared at 350 µM concentration. Bruker *T*_1_, *T*_2_ (standard)^68^, and (^1^H)-^15^N heteronuclear NOE (non-standard) pulse sequences were implemented^68^. The latter was modified to remove anti-TROSY components and produce cleaner TROSY. Data were collected as pseudo-3D experiments with temperature compensation (See Supplementary Methods for further details about parameters). Sample stability prior and post backbone dynamics experiments was assessed by acquiring ^1^H-^15^N TROSY-HSQC spectra.

### Data availability

The data that support the findings of this study are available from the corresponding author upon request.

## Electronic supplementary material

Supplementary Information
