# Supplementary material for: Survey of solution dynamics in Src kinase reveals allosteric cross talk between the ligand binding and regulatory sites
Source: Nat Commun. 2017 Dec 18;8:2160. doi: 10.1038/s41467-017-02240-6 (PMC5735167; doi:10.1038/s41467-017-02240-6)
Supplement: Supplementary file 1 — Supplementary Information [file 41467_2017_2240_MOESM1_ESM.pdf]

Supplementary information

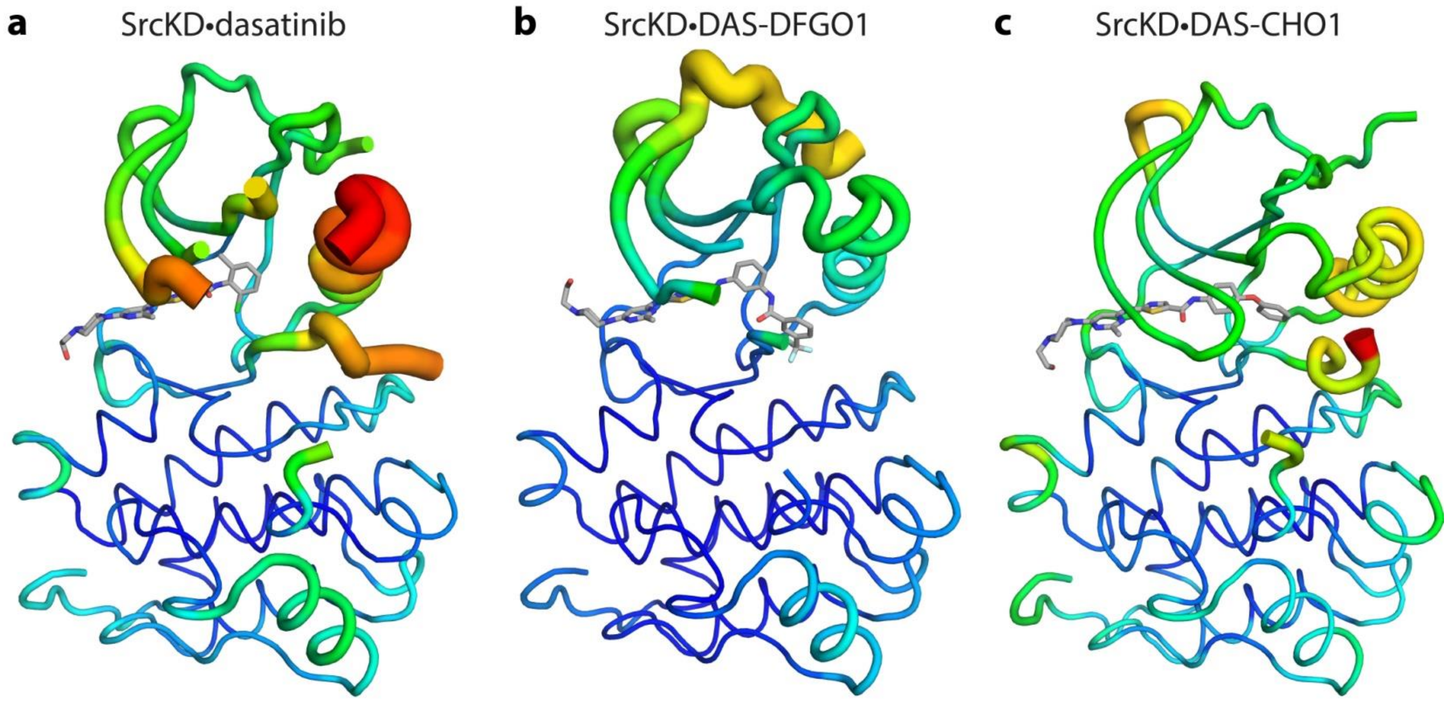

Supplementary Figure 1 | Minimal differences are observed in the C-lobe conformation and dynamics of SrcKD crystal structures bound to conformation-selective ligands. The B-factors of the SrcKD crystal structures in complex with **(a)** dasatinib, **(b)** DAS-DFGO1, **(c)** DAS-CHO1, are shown to infer about their backbone dynamics. The greater the thickness of the cartoon tube the greater the extent of flexibility. In addition, regions colored in red, orange, yellow, green, cyan and blue, also reflect the extent of flexibility ranging from high (red) to low (blue) respectively. Here DAS-DFGO1 and DAS-CHO1, which lack the phenyl moiety methyl group in the DAS-DFGO2 and DAS-CHO2 variants, were used in these crystal structures[1]

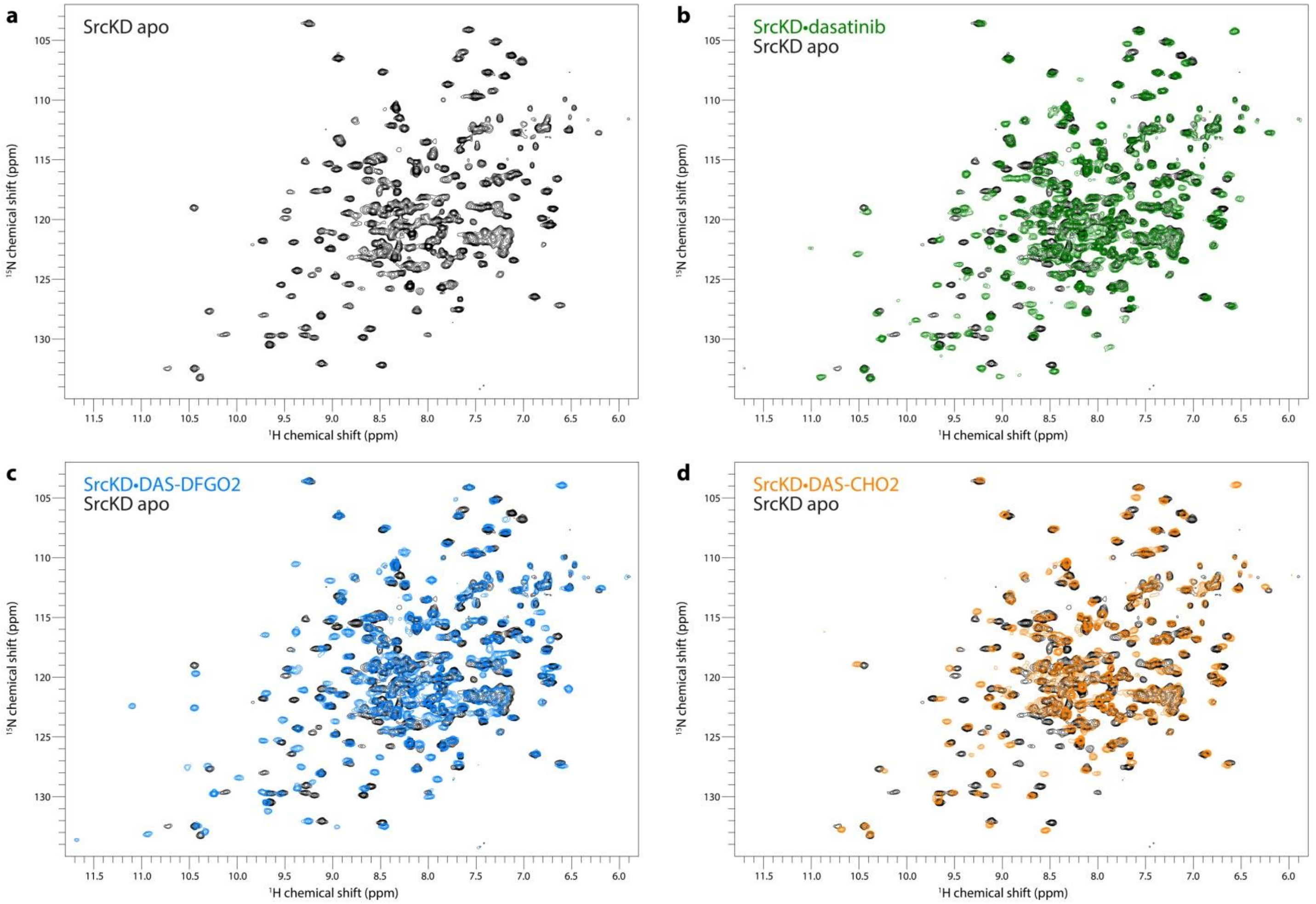

Supplementary Figure 2 | ^{1}H-^{15}N TROSY-HSQC spectra of apo SrckD and distinct ligand stabilized active and inactive states. Spectral comparison of **(a)** apo SrcKD, with ligand bound states: **(b)** dasatinib, **(c)** DAS-DFGO2, and **(d)** DAS-CHO2, indicate that the ligands induce distinct backbone amide resonance CSPs and IPs. These perturbations include the appearance of new resonances that were previously broadened into intermediate exchange in the apo state. Resonance intensities within each state are also heterogeneous, suggesting different dynamics across the backbone.

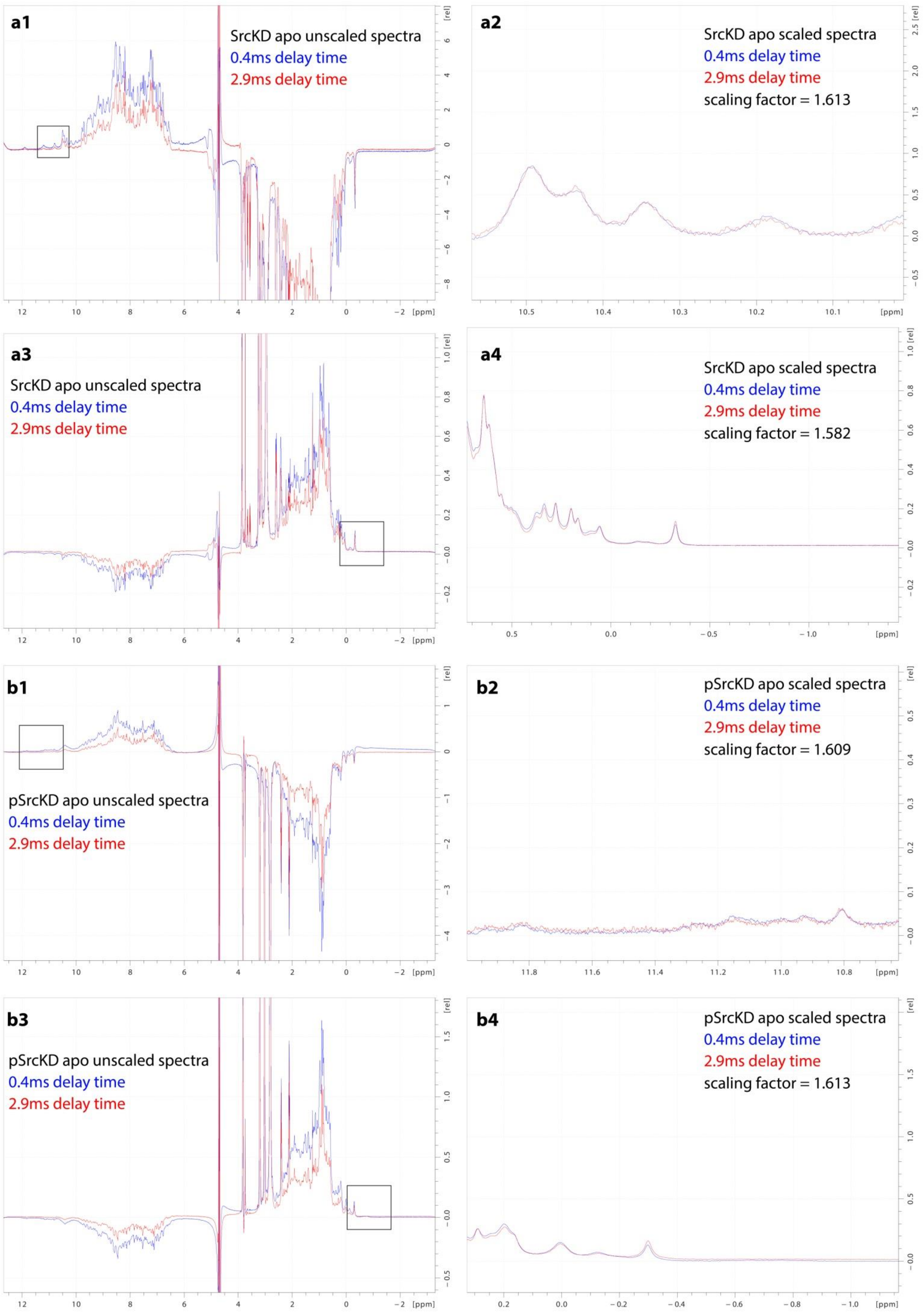
a1
SrcKD apo unscaled spectra
0.4ms delay time
2.9ms delay time
a2
SrcKD apo scaled spectra
0.4ms delay time
2.9ms delay time
scaling factor = 1.613
a3
SrcKD apo unscaled spectra
0.4ms delay time
2.9ms delay time
a4
SrcKD apo scaled spectra
0.4ms delay time
2.9ms delay time
scaling factor = 1.582
b1
pSrcKD apo unscaled spectra
0.4ms delay time
2.9ms delay time
b2
pSrcKD apo scaled spectra
0.4ms delay time
2.9ms delay time
scaling factor = 1.609
b3
pSrcKD apo unscaled spectra
0.4ms delay time
2.9ms delay time
b4
pSrcKD apo scaled spectra
0.4ms delay time
2.9ms delay time
scaling factor = 1.613
[ppm]
[rel]

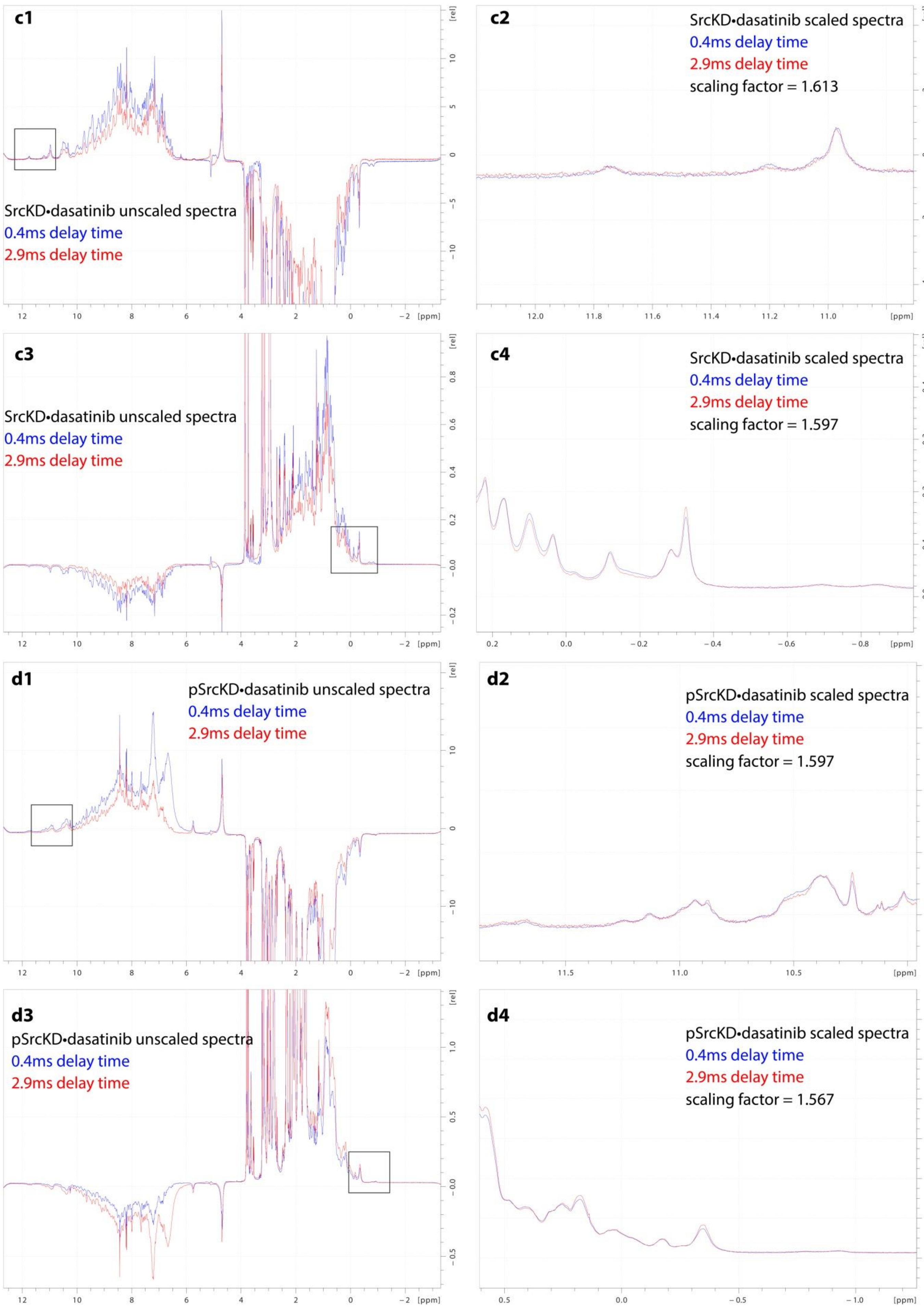
c1
SrcKD•dasatinib unscaled spectra
0.4ms delay time
2.9ms delay time
c2
SrcKD•dasatinib scaled spectra
0.4ms delay time
2.9ms delay time
scaling factor = 1.613
c3
SrcKD•dasatinib unscaled spectra
0.4ms delay time
2.9ms delay time
c4
SrcKD•dasatinib scaled spectra
0.4ms delay time
2.9ms delay time
scaling factor = 1.597
d1
pSrcKD•dasatinib unscaled spectra
0.4ms delay time
2.9ms delay time
d2
pSrcKD•dasatinib scaled spectra
0.4ms delay time
2.9ms delay time
scaling factor = 1.597
d3
pSrcKD•dasatinib unscaled spectra
0.4ms delay time
2.9ms delay time
d4
pSrcKD•dasatinib scaled spectra
0.4ms delay time
2.9ms delay time
scaling factor = 1.567
[ppm]
[rel]

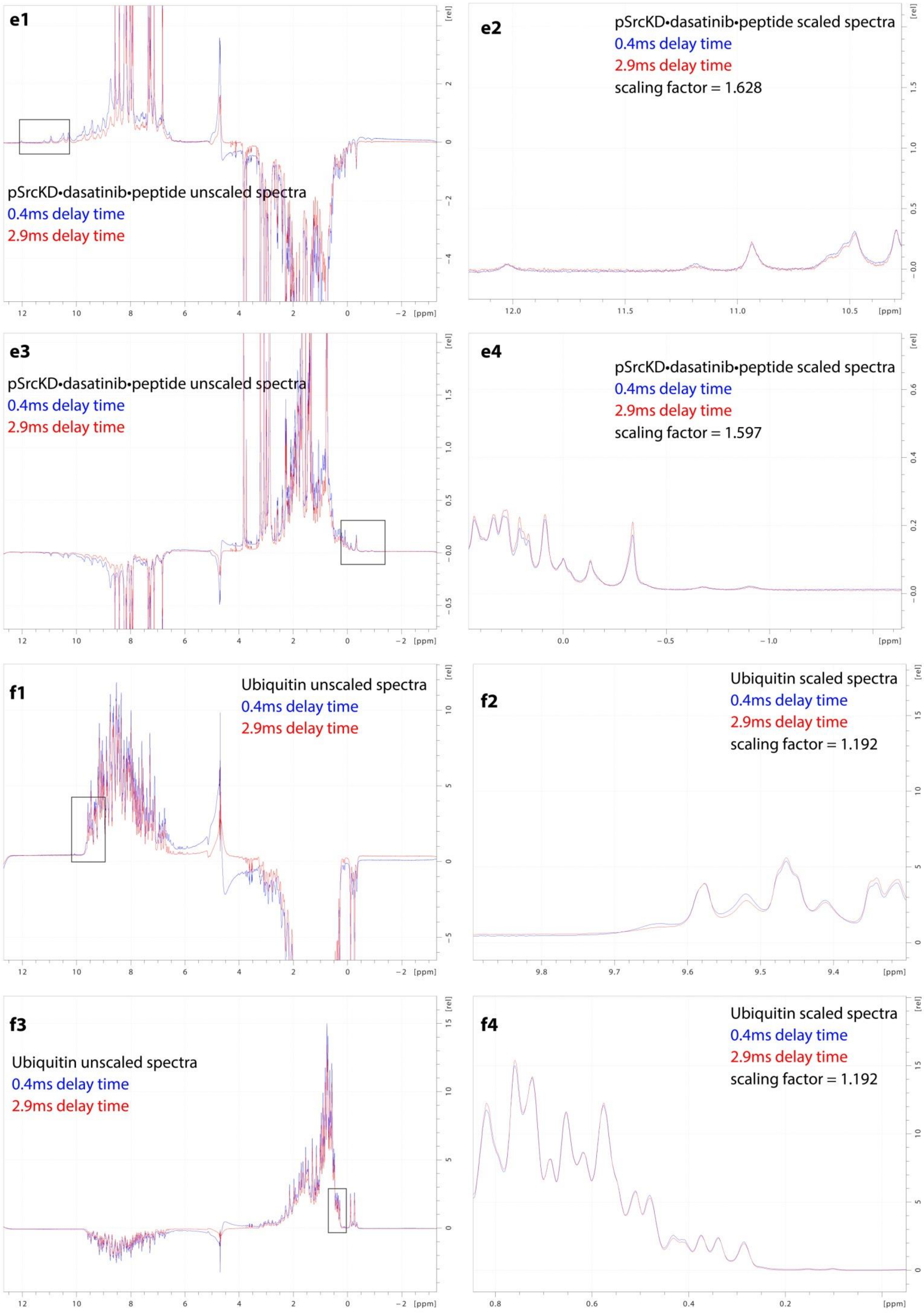
e1
pSrcKD•dasatinib•peptide unscaled spectra
0.4ms delay time
2.9ms delay time
e2
pSrcKD•dasatinib•peptide scaled spectra
0.4ms delay time
2.9ms delay time
scaling factor = 1.628
e3
pSrcKD•dasatinib•peptide unscaled spectra
0.4ms delay time
2.9ms delay time
e4
pSrcKD•dasatinib•peptide scaled spectra
0.4ms delay time
2.9ms delay time
scaling factor = 1.597
f1
Ubiquitin unscaled spectra
0.4ms delay time
2.9ms delay time
f2
Ubiquitin scaled spectra
0.4ms delay time
2.9ms delay time
scaling factor = 1.192
f3
Ubiquitin unscaled spectra
0.4ms delay time
2.9ms delay time
f4
Ubiquitin scaled spectra
0.4ms delay time
2.9ms delay time
scaling factor = 1.192
[rel]
[ppm]

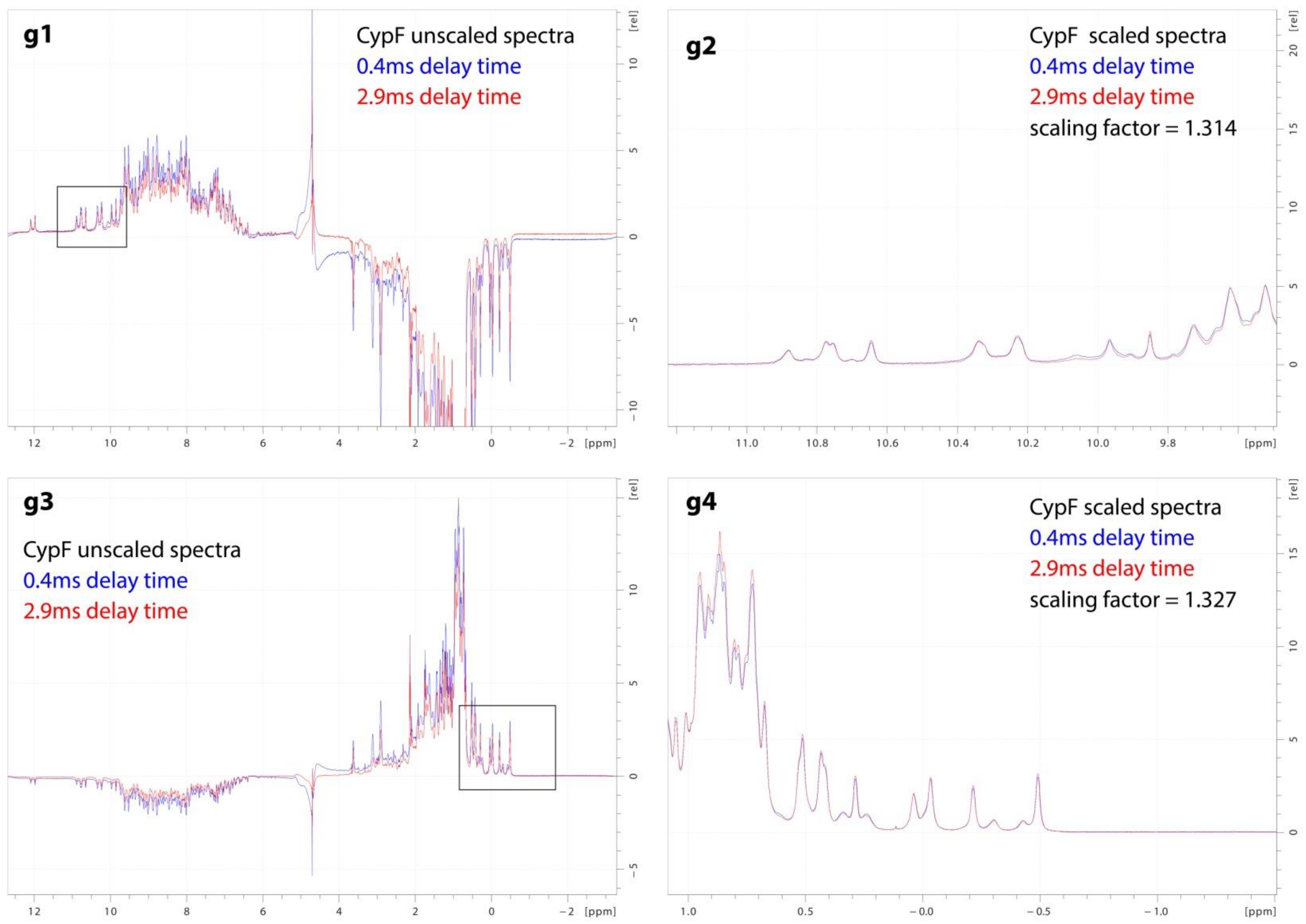

Supplementary Figure 3 | Molecular weight evaluation of distinct SrcKD states using a jump-and-return spin echo type pulse sequence indicates monomeric behavior. (a-e) Representative unscaled and scaled spectra for different SrcKD states and positive controls Ubiquitin **(f)**, and Cyclophilin F **(g)** are shown for the amide and methyl region at two different delay times. The spectrum with the long delay time (red) is compared to its corresponding short delay time spectrum (blue), and indicates resonances with an overall reduction in intensity due to T_2 relaxation **(a-e)**. The long delay time spectrum is scaled up for non-sharp resonances in the amide and methyl region until its intensity is similar to the short delay time spectrum. The scaling factor was used to calculate the molecular weight of the protein sample (See Supplementary Table. 2 for relevant parameters and Supplementary Methods for details). Stable monomeric proteins Ubiquitin **(f)** and Cyclophilin F **(g)** were used as positive controls. ^{1}H chemical shifts and relative intensity are shown on the x and y axis respectively.

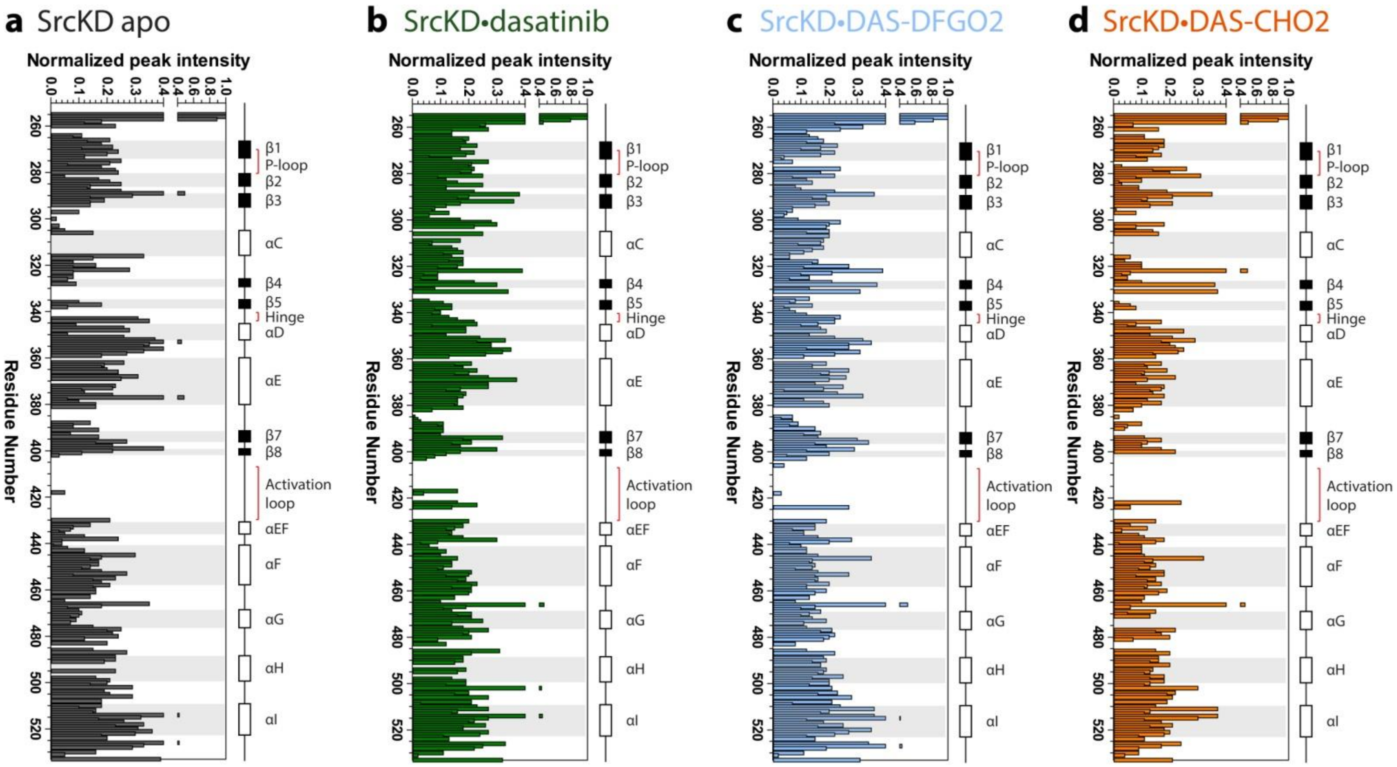

Supplementary Figure 4 | Normalized resonance intensities of apo SrcKD and ligand-bound states. (a-d) Distinct SrcKD states show a heterogeneous distribution of resonance intensities indicative of a backbone varying in flexibility and rigidity. High resonance intensities for example at the N-terminus suggest that fast internal motions have a more pronounced contribution to their overall intensities than broadening from slow internal motions (intermediate to slow conformational exchange). Conversely, unobservable resonances (intermediate conformational exchange), or low intensities suggest the opposite.

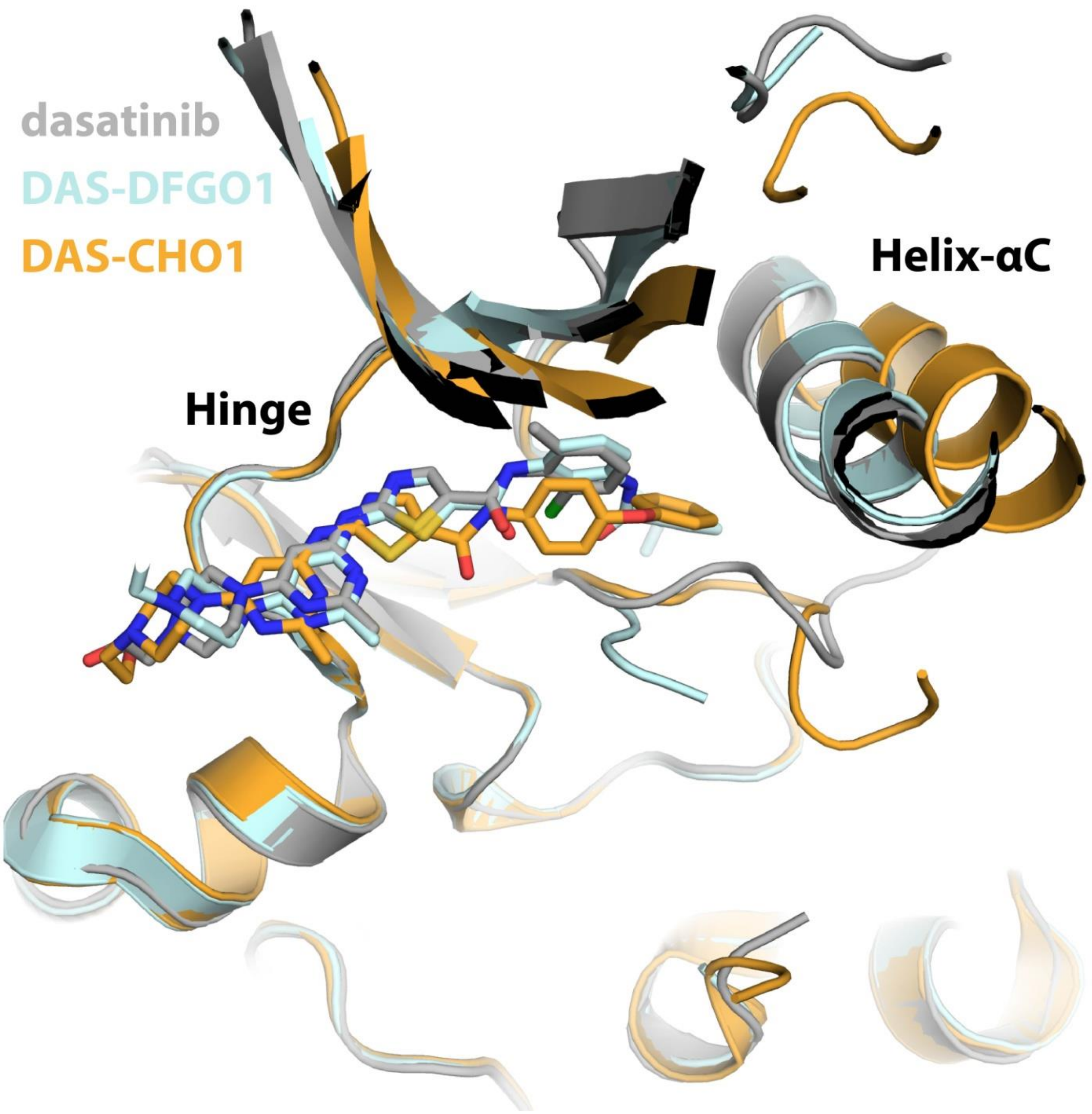

Supplementary Figure 5 | The binding mode of DAS-CHO1 is shifted across the hinge compared to dasatinib and DAS-DFGO1. Crystal structure alignment of SrcKD dasatinib, DAS-DFGO1, and DAS-CHO1 (PDB: 3G5D, 4YBK, 4YBJ)[1, 2] inhibitor complexes reveals that the binding mode of DAS-CHO1 differs slightly across the hinge region despite a common scaffold moiety shared between all ligands. We expect the binding modes for DAS-DFGO2 and DAS-CHO2 to be similar to their respective DAS-DFGO1 and DAS-CHO1 variants because DAS-DFGO2 and DAS-CHO2 variants only differ in having a methyl group on the phenyl moiety.

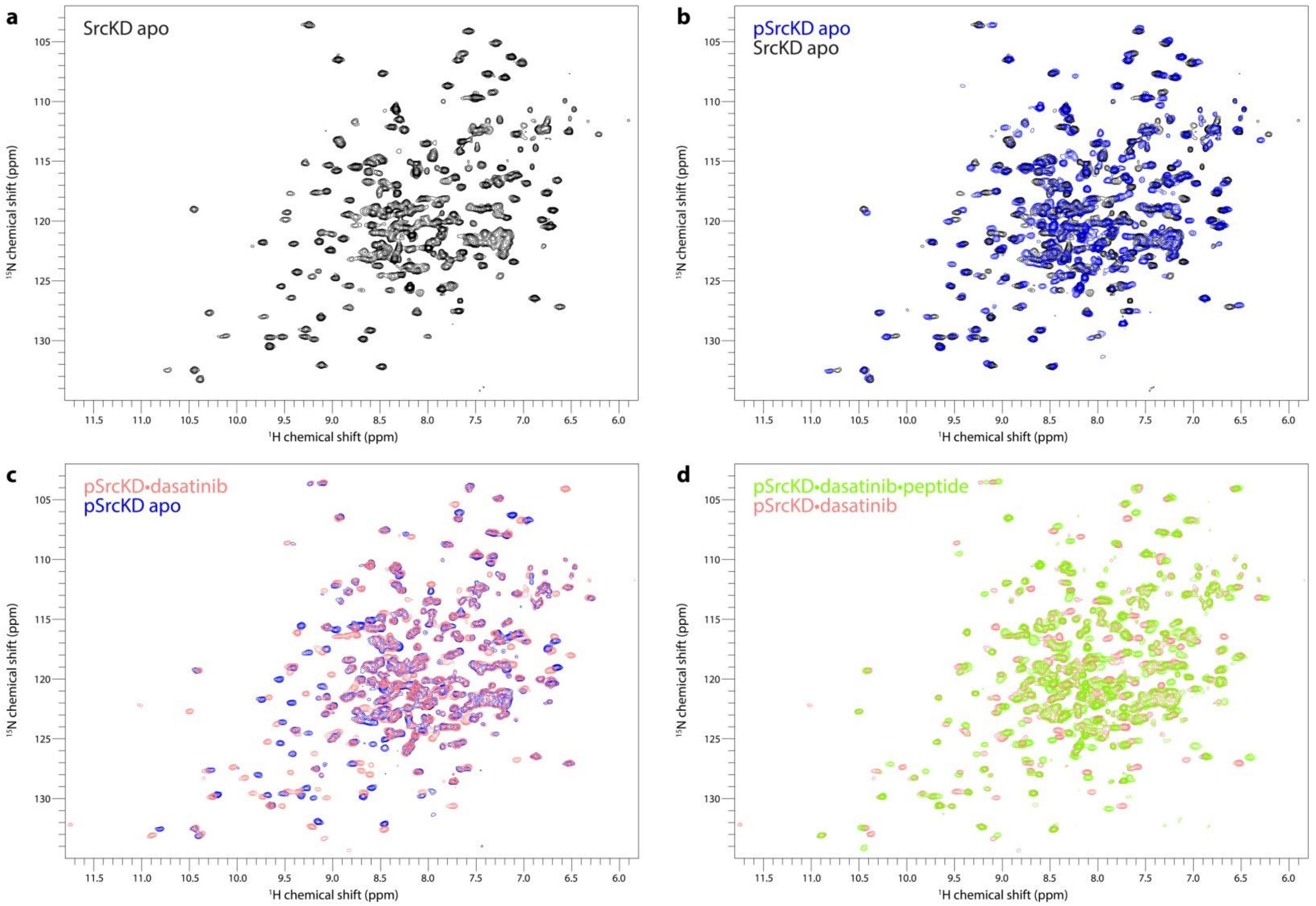

Supplementary Figure 6 | ^{1}H-^{15}N TROSY-HSQC spectra of the SrcKD with different active conformation inputs. Spectra of **(a)** apo SrcKD were compared with the spectra of apo SrcKD after perturbation with different active conformation stabilizing inputs: **(b)** activation loop autophosphorylation, **(c)** plus dasatinib, **(d)** plus substrate peptide binding. Overall each input gives rise to different backbone amide resonance CSPs and IPs indicative of their distinct perturbation sites and mechanism of stabilizing the active conformation. Activation loop autophosphorylation induces resonance splitting, suggesting two-state slow NMR time scale conformational exchange which largely persists following dasatinib and substrate peptide binding.

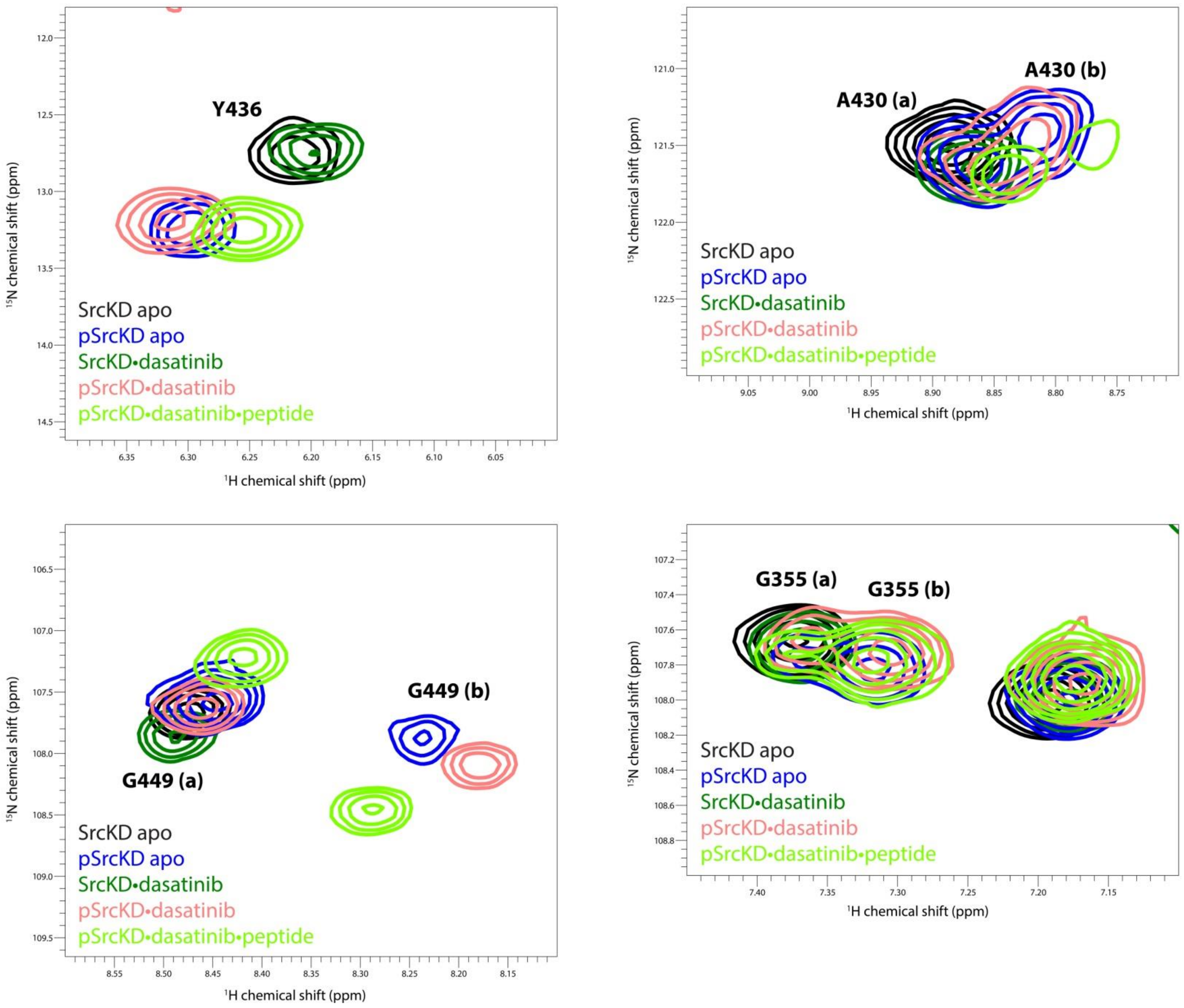

Supplementary Figure 7 | Autophosphorylation results in CSPs and resonance splitting which can persist in the presence of dasatinib and substrate peptide. Selected examples of resonances (Gly355, Ala430, Tyr436 Gly449) undergoing autophosphorylation dependent CSP, while some also undergo additional resonance splitting indicative of two state slow conformational exchange.

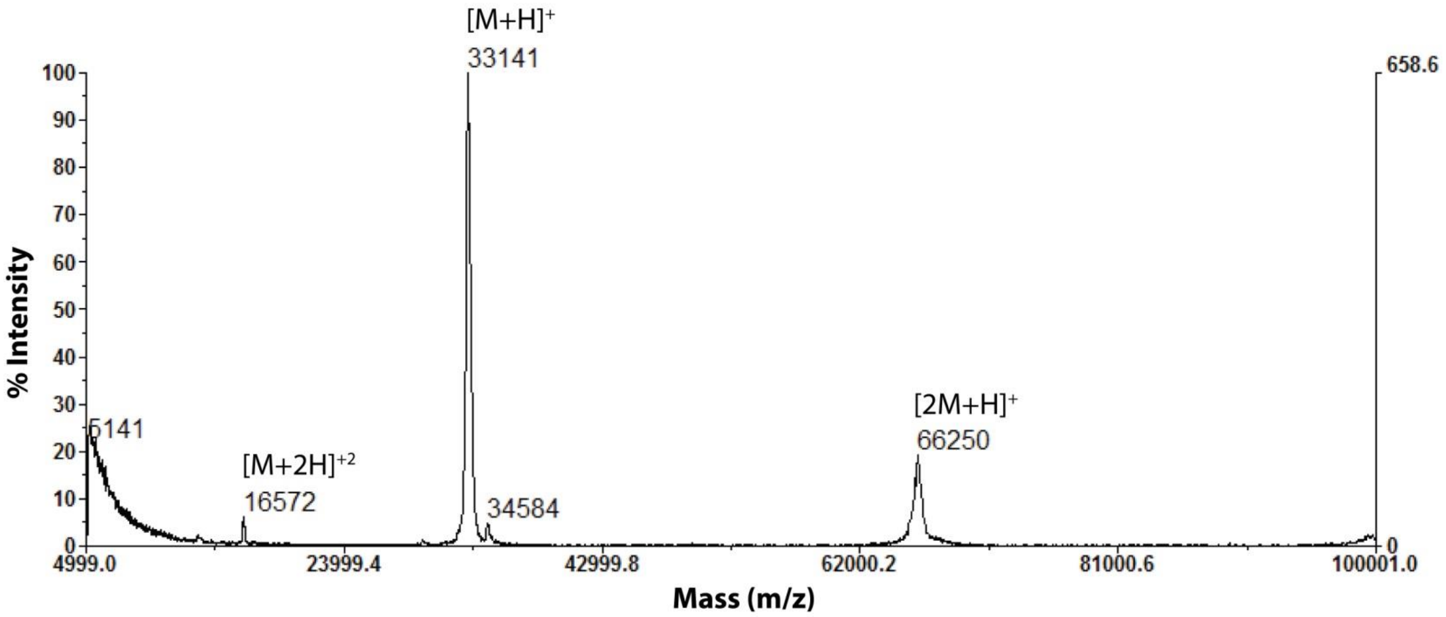

Supplementary Figure 8 | MALDI-TOF spectrum of ^{15}N-labeled pSrcKD suggests a single phosphorylated species. The single peak $[M+H]^+$ with a mass/z of 33141 shows that pSrcKD is a predominantly single phosphorylated species. Subsequent LCMS analysis indicated that Y416 was the main phosphorylation site because the peptide LIEDNEYTAR was found to be phosphorylated 90% of the time.

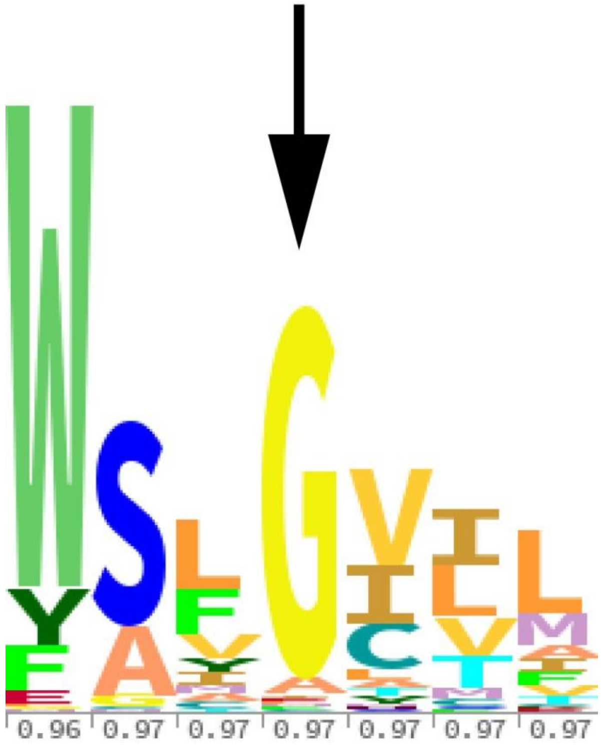

Supplementary Figure 9 | Hidden markov model logos of all human protein kinase domain sequences spanning the helix-αF region indicates that Gly499 is highly conserved. Gly449 corresponds to the Gly residue denoted by the arrow. This Gly has the largest height in the stack indicating that it is highly conserved in human protein kinases at this position in the sequence alignment. The height of the letter stack corresponds to a measure of the invariance, whilst the height of each letter within the stack reflects the frequency of that amino acid for that position in the sequence alignment. Numbers below the stacks correspond to the occupancy, which describes the probability of observing an amino acid shown from the stack. The HMM logo was generated using skylign[3].

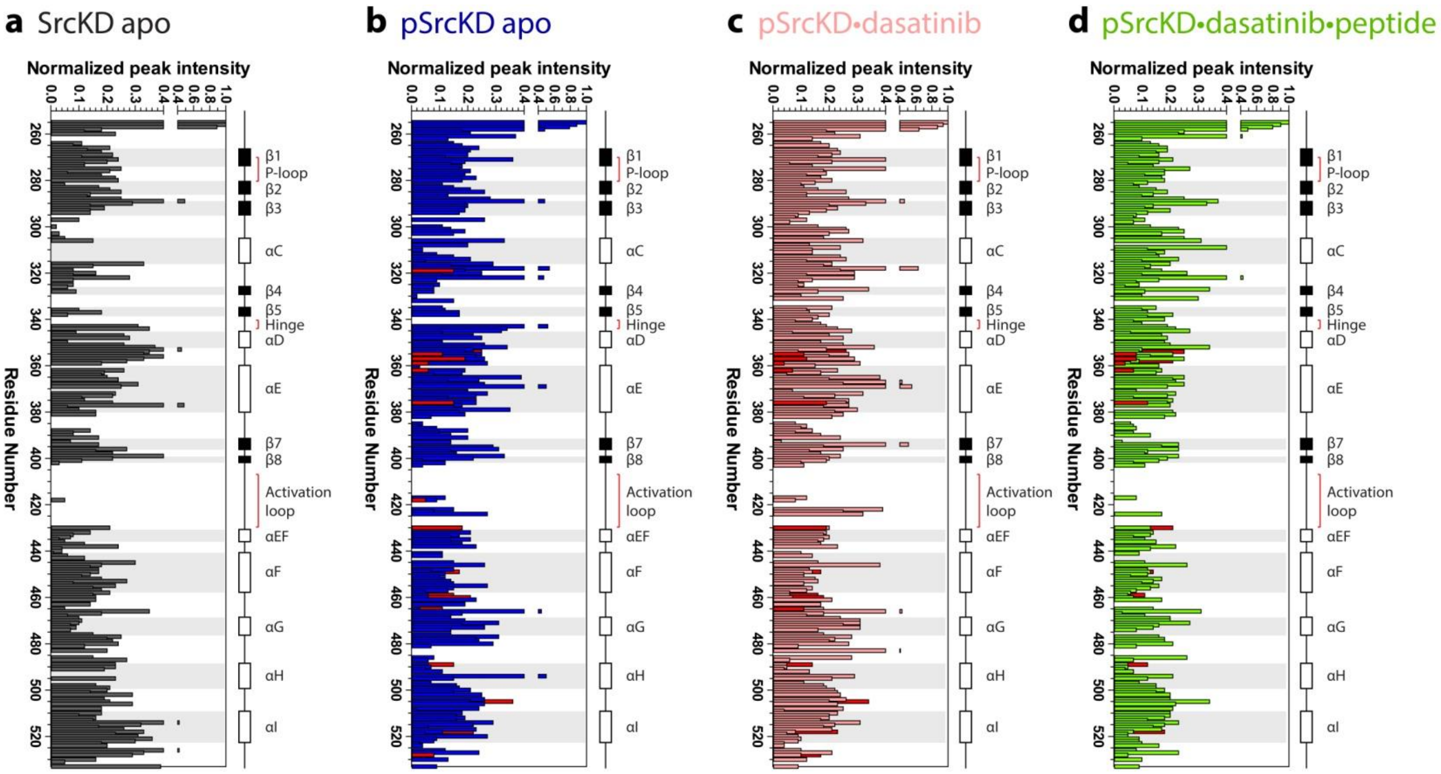

Supplementary Figure 10 | Normalized resonance intensities of SrcKD states with distinct active inputs. (a-d) Distinct SrcKD states show a heterogeneous distribution of resonance intensities indicative of a flexible backbone. High resonance intensities for example at the N-terminus suggest that fast internal motions have a more pronounced contribution to their overall intensities than broadening from slow internal motions (intermediate to slow conformational exchange). Conversely, unobservable resonances (intermediate conformational exchange), or low intensities suggest the opposite. Red bars indicate the intensity of the second resonance that arises from resonance splitting following activation loop autophosphorylation.

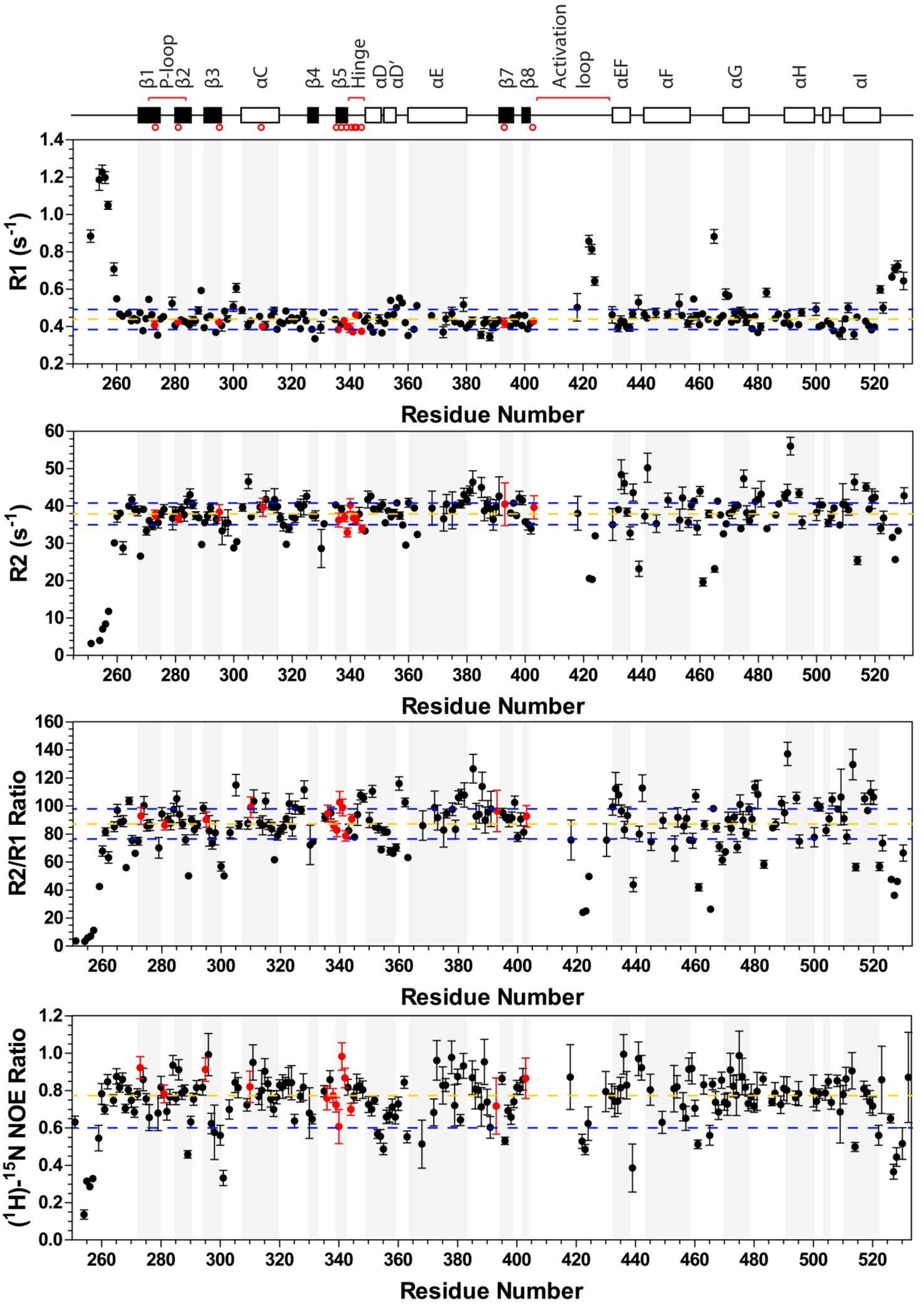

β1
P-loop
β2
β3
αC
β4
β5
Hinge
αD
αD'
αE
β7
β8
Activation loop
αEF
αF
αG
αH
αI
R1 (s-1)
Residue Number
R2 (s-1)
Residue Number
R2/R1 Ratio
Residue Number
(1H)-15N NOE Ratio
Residue Number

Supplementary Figure 11 | Fast backbone dynamics of the pSrcKD•dasatinib state. Results for R_1, R_2, R_2:R_1 ratio, and heteronuclear NOE ratio are plotted along with the backbone secondary structure. The R_2:R_1 ratio analysis is an indicator of the global tumbling. Data points colored in red and red circles under the secondary structure schematic indicate residues which directly interact with dasatinib. Residues which reflect R_1, R_2, and R_2:R_1 values within one standard deviation of the trimmed mean suggests that they behave rigidly and only have significant motion attributed to global tumbling. Hallmarks of enhanced fast internal motion include: $R_{1,}$ one standard deviation greater than the trimmed mean, and R_2 and R_2:R_1 values one standard deviation less than the trimmed mean. Conversely, R_2 and R_2:R_1 ratios values one standard deviation above the trimmed mean, are indicative of conformational exchange. Residues with heteronuclear NOE ratios below 0.6 suggests that they possess fast internal motion. Evidently fast and slow internal motions are dispersed throughout the backbone with some regions showing both types of motion such as the R_2:R_1 values around the carboxy-terminus. See Supplementary Methods for details on error bar determination.

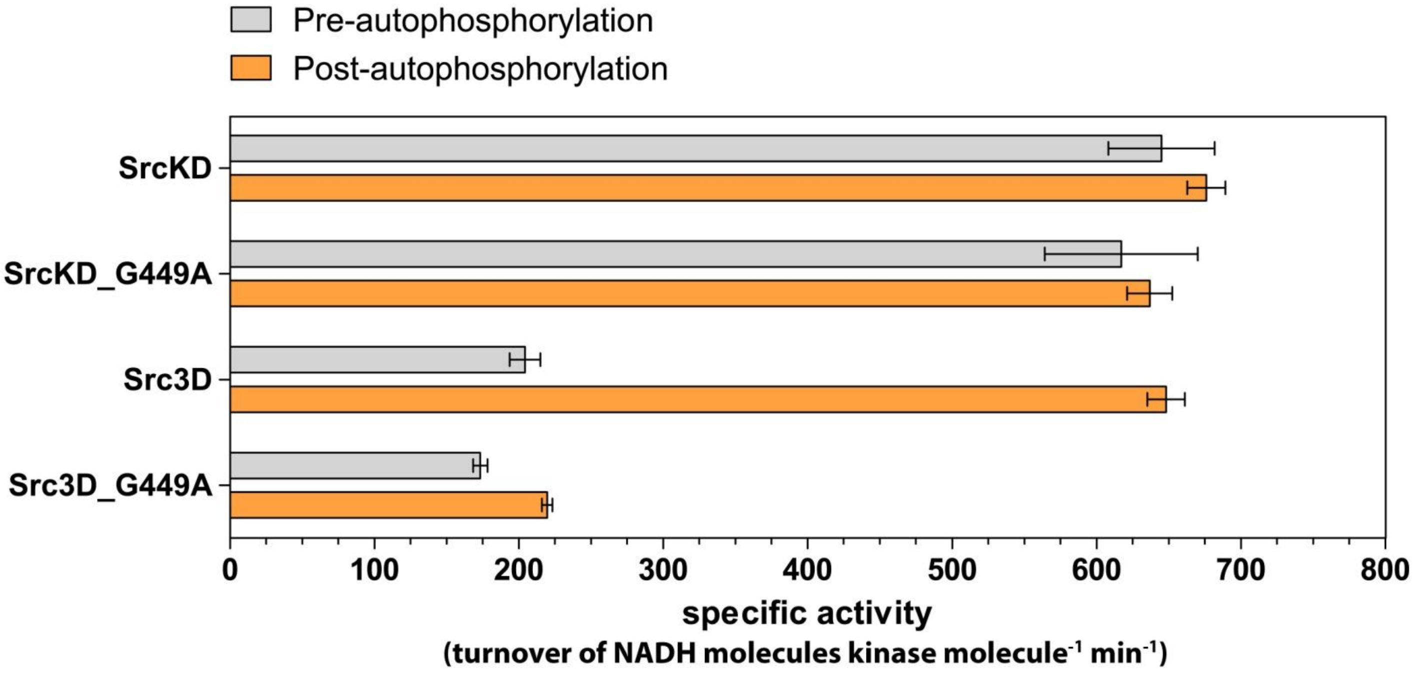

Supplementary Figure 12 | Gly449 is implicated in mediating activation loop autophosphorylation dependent maximal kinase activity in Src3D. The specific activities for wild type and G449A mutants of SrcKD and Src3D, against substrate peptide, pre and post-autophosphorylation are shown. In the non-phosphorylated state, the basal specific activities of Src3D and Src3D_G449A, are similar, but post-autophosphorylation differences become apparent because the fold increase in specific activity is 3-fold greater for Src3D relative to non-phosphorylated, whereas for Src3D_G449A there is only a 1.3-fold increase. In contrast SrcKD, and SrcKD_G449A maximal activities are independent of autophosphorylation and are at comparable levels. This suggests that Gly449 is important in mediating the activation of near full length Src (minus the SH4 domain) in an autophosphorylation dependent manner. The error bars are based on the s.d of specific activity values determined from experiments conducted in triplicate or duplicate.

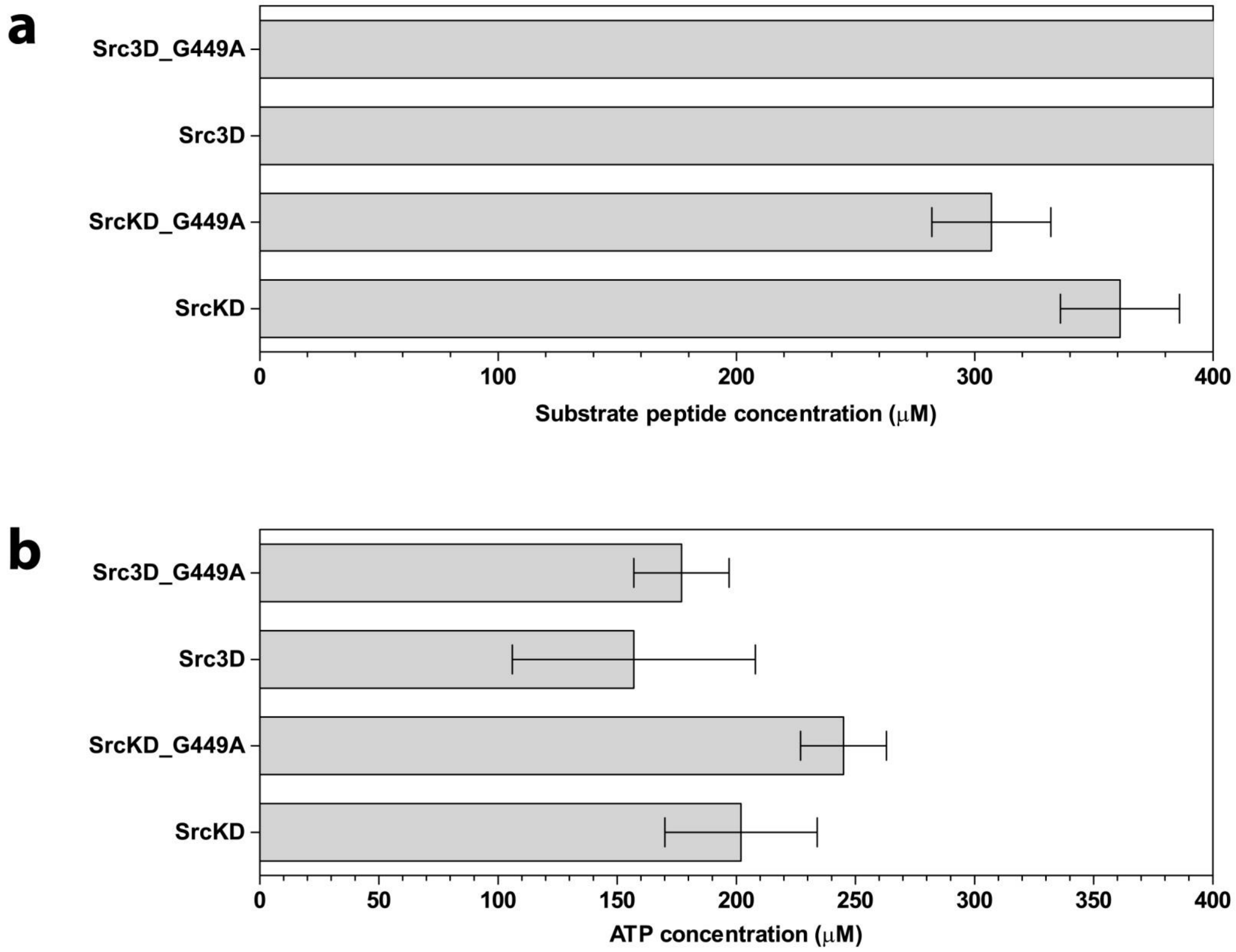

Supplementary Figure 13 | *Km* peptide and *Km* ATP are similar for wild type and G449A in the context of Src3D and SrcKD suggesting that G449A in the center of helix-αF does not significantly impact substrate binding affinity. (a) K_m peptide determination at 0.4 mM ATP. The K_m peptide values for Src3D, and Src3D_G449A, were greater than 500 μM. **(b)** K_m ATP determination at 0.8 mM substrate peptide. The error bars are based on the s.d of K_m values determined from experiments conducted in triplicate or duplicate.

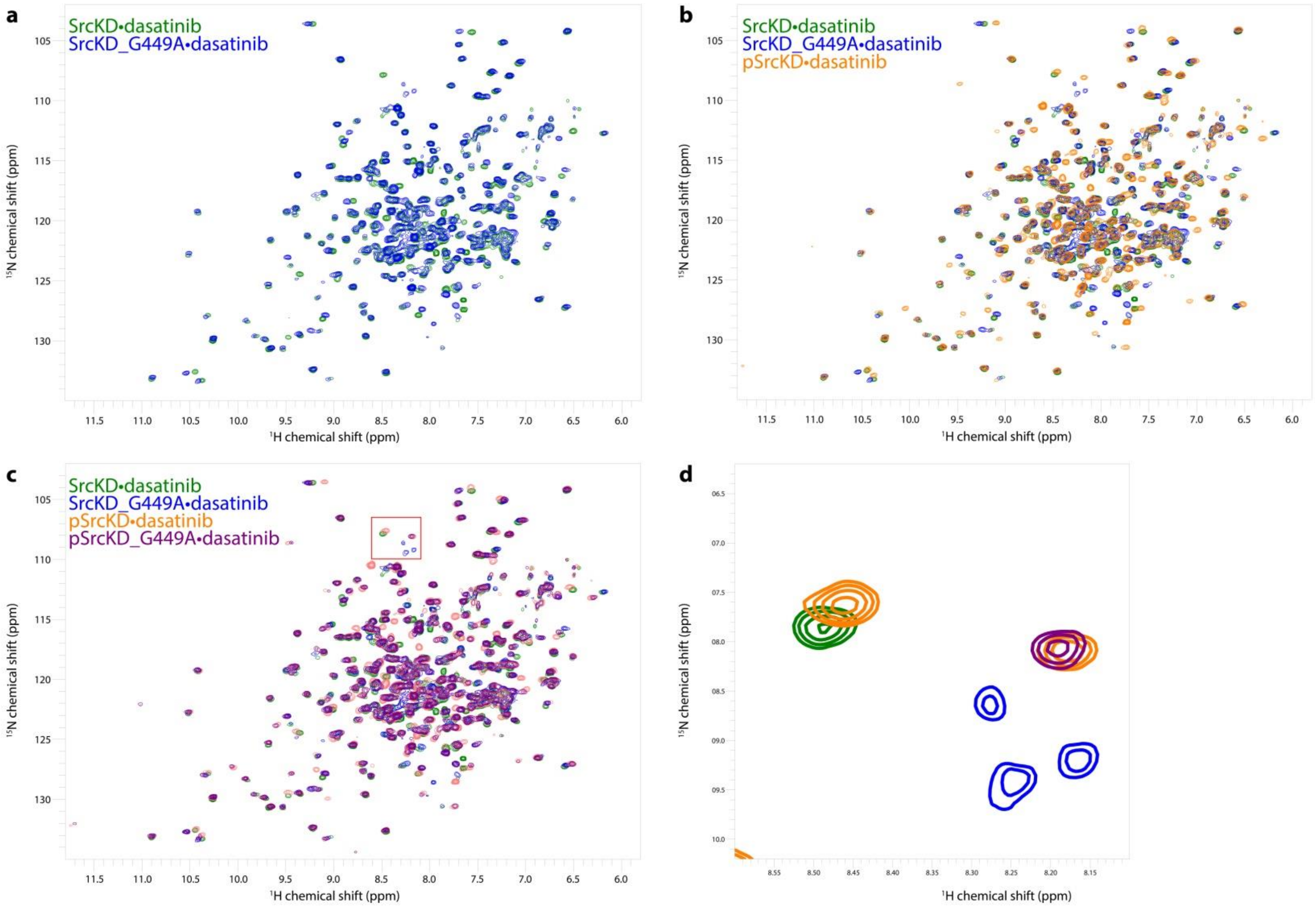

Supplementary Figure 14 | G449A induces distinct CSPs in the SrcKD_G449A•dasatinib and pSrcKD_G449A•dasatinib states compared to wild type suggesting that it perturbs CSPs induced by dasatinib binding and activation loop autophosphorylation. (a-c) ^{1}H-^{15}N TROSY spectra overlay of the wild type and G449A states shows wide spread CSPs. **(d)** Zoomed in region of the spectra where the Gly449 resonance occurs. In the SrcKD_G449A dasatinib state, the resonance corresponding to Gly449 as seen in the wild type is missing either because it adopts a new chemical environment or has broadened out into intermediate conformational exchange or both. Interestingly upon activation loop autophosphorylation of SrcKD_G449A to yield pSrcKD_G449A, a new unassigned resonance emerges, and appears to adopt a similar chemical environment to the wild type Gly449 resonance.

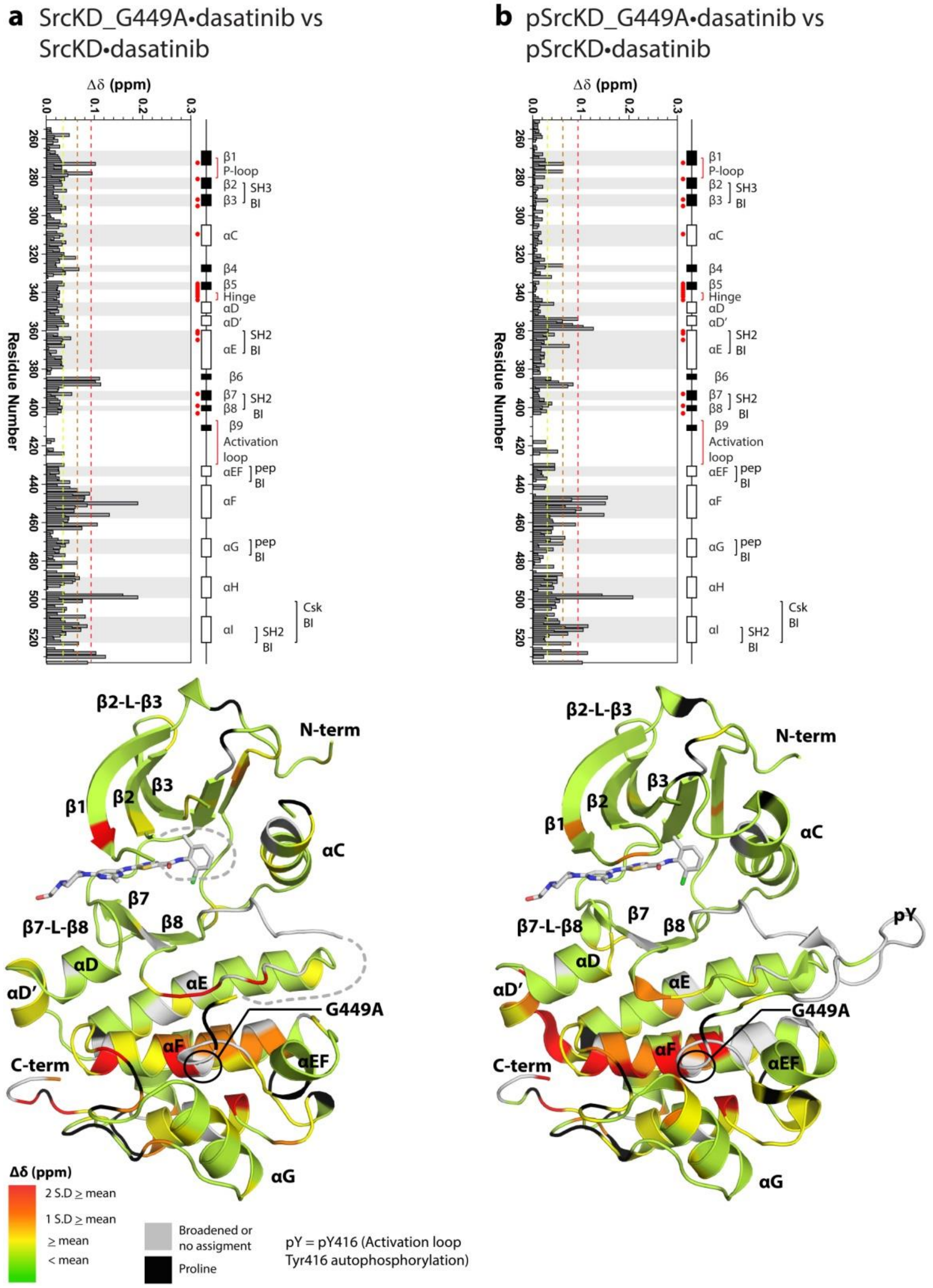

a SrcKD_G449A•dasatinib vs SrcKD•dasatinib
b pSrcKD_G449A•dasatinib vs pSrcKD•dasatinib
Δδ (ppm)
0.0
0.1
0.2
0.3
Residue Number
β1
P-loop
β2
β3
SH3
BI
αC
β4
β5
Hinge
αD
αD′
αE
SH2
BI
β6
β7
β8
SH2
BI
β9
Activation loop
αEF
pep
BI
αF
αG
pep
BI
αH
αI
SH2
BI
Csk
BI
β2-L-β3
N-term
β7-L-β8
G449A
C-term
pY
Δδ (ppm)
2 S.D ≥ mean
1 S.D ≥ mean
≥ mean
< mean
Broadened or no assigment
Proline
pY = pY416 (Activation loop Tyr416 autophosphorylation)

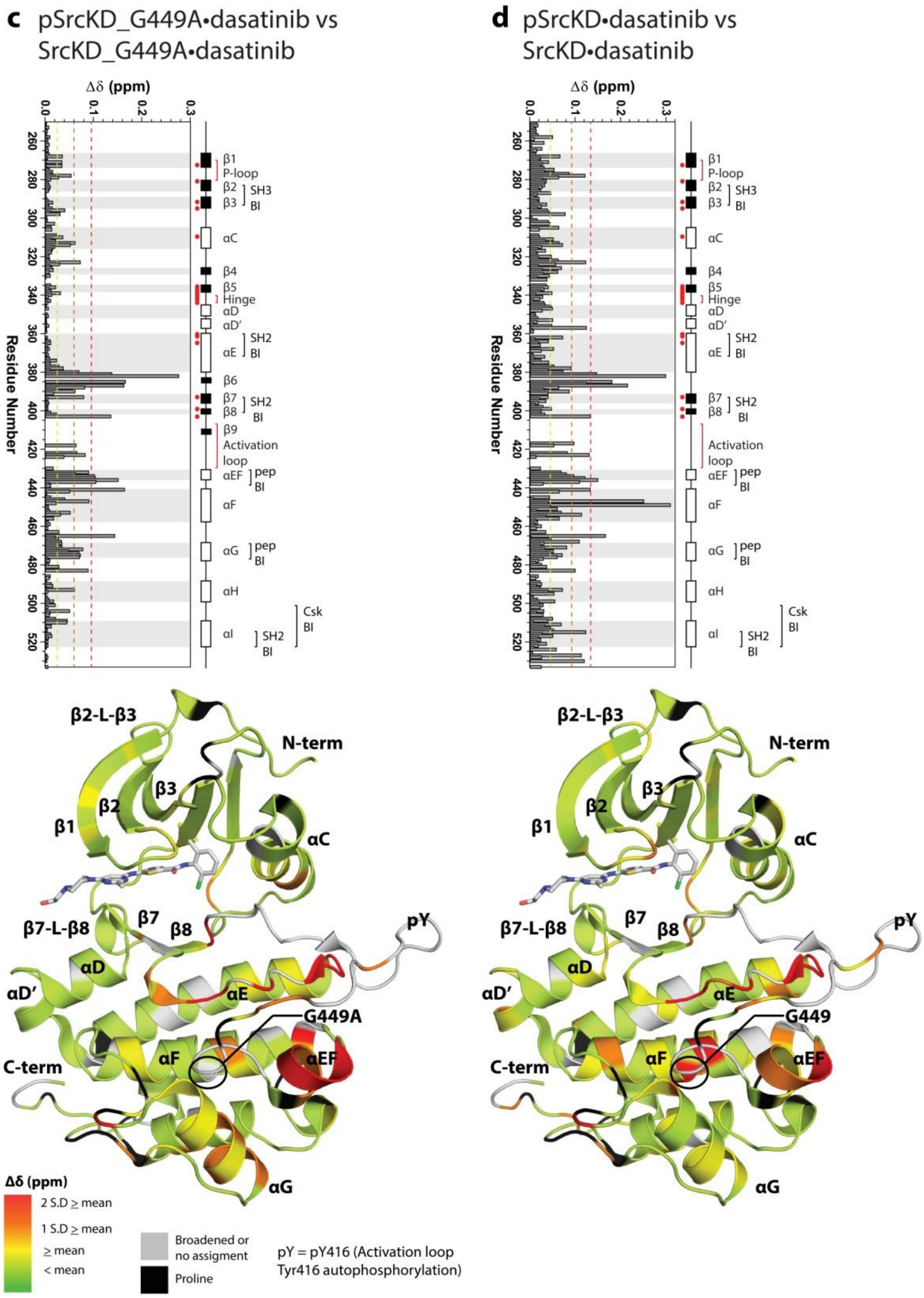

Supplementary Figure 15 | G449A induces CSPs that largely affect the local C-lobe and reduces the magnitude of perturbation in helix-αF upon activation loop autophosphorylation. CSPs between G449A and wild type in the context of **(a)** SrcKD•dasatinib and **(b)** pSrcKD•dasatinib were analyzed by histograms and CSP structure mapping to probe the conformational changes induced by the mutant. In the unphosphorylated state **(a)**, CSPs occur largely around helix-αF, where G449A resides, and similarly in the phosphorylated state **(b)**, but here the magnitude of CSPs are greater. **(c)** CSPs between pSrcKD_G449A•dasatinib and SrcKD_G449A•dasatinib were also analyzed to probe the effect of activation loop autophosphorylation in the context of G449A and

compared to **(d)** wild type. Strong CSPs occur around helix-αEF in both **(c, d)** but additional strong perturbations occur within the center of helix-αF for **(d)** wild type. This indicates that G449A attenuates the crosstalk that occurs between helix-αF and the activation loop upon autophosphorylation of SrcKD•dasatinib.

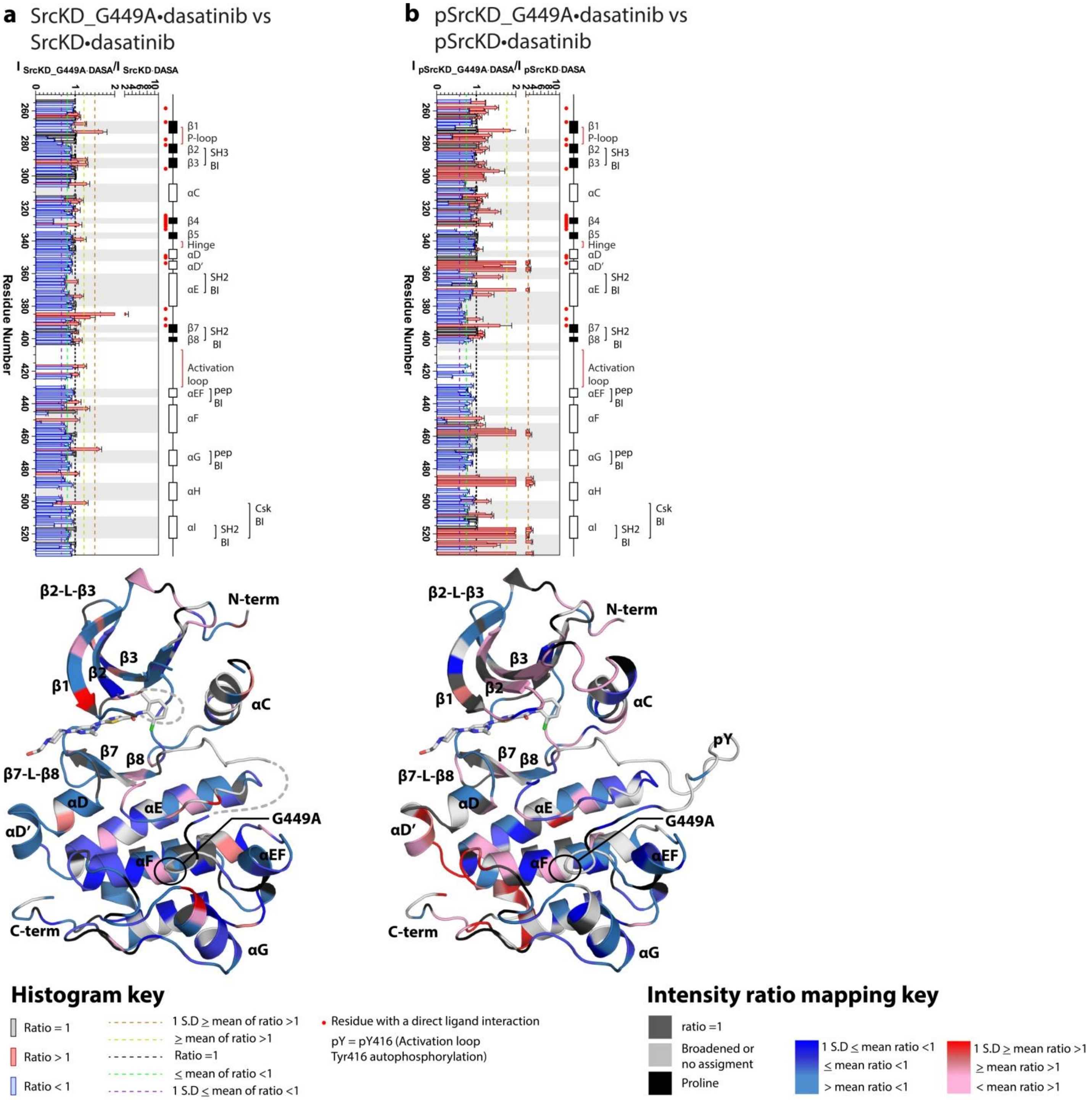
a SrcKD_G449A•dasatinib vs SrcKD•dasatinib
b pSrcKD_G449A•dasatinib vs pSrcKD•dasatinib
Residue Number
β1
P-loop
β2
β3
SH3 BI
αC
β4
β5
Hinge
αD
αD′
αE
SH2 BI
β7
β8
Activation loop
αEF
pep BI
αF
αG
αH
αI
Csk BI
β2-L-β3
N-term
β7-L-β8
G449A
C-term
pY
Histogram key
Ratio = 1
Ratio > 1
Ratio < 1
1 S.D ≥ mean of ratio >1
≥ mean of ratio >1
Ratio =1
≤ mean of ratio <1
1 S.D ≤ mean of ratio <1
Residue with a direct ligand interaction
pY = pY416 (Activation loop Tyr416 autophosphorylation)
Intensity ratio mapping key
ratio =1
Broadened or no assigment
Proline
1 S.D ≤ mean ratio <1
≤ mean ratio <1
> mean ratio <1
1 S.D ≥ mean ratio >1
≥ mean ratio >1
< mean ratio >1

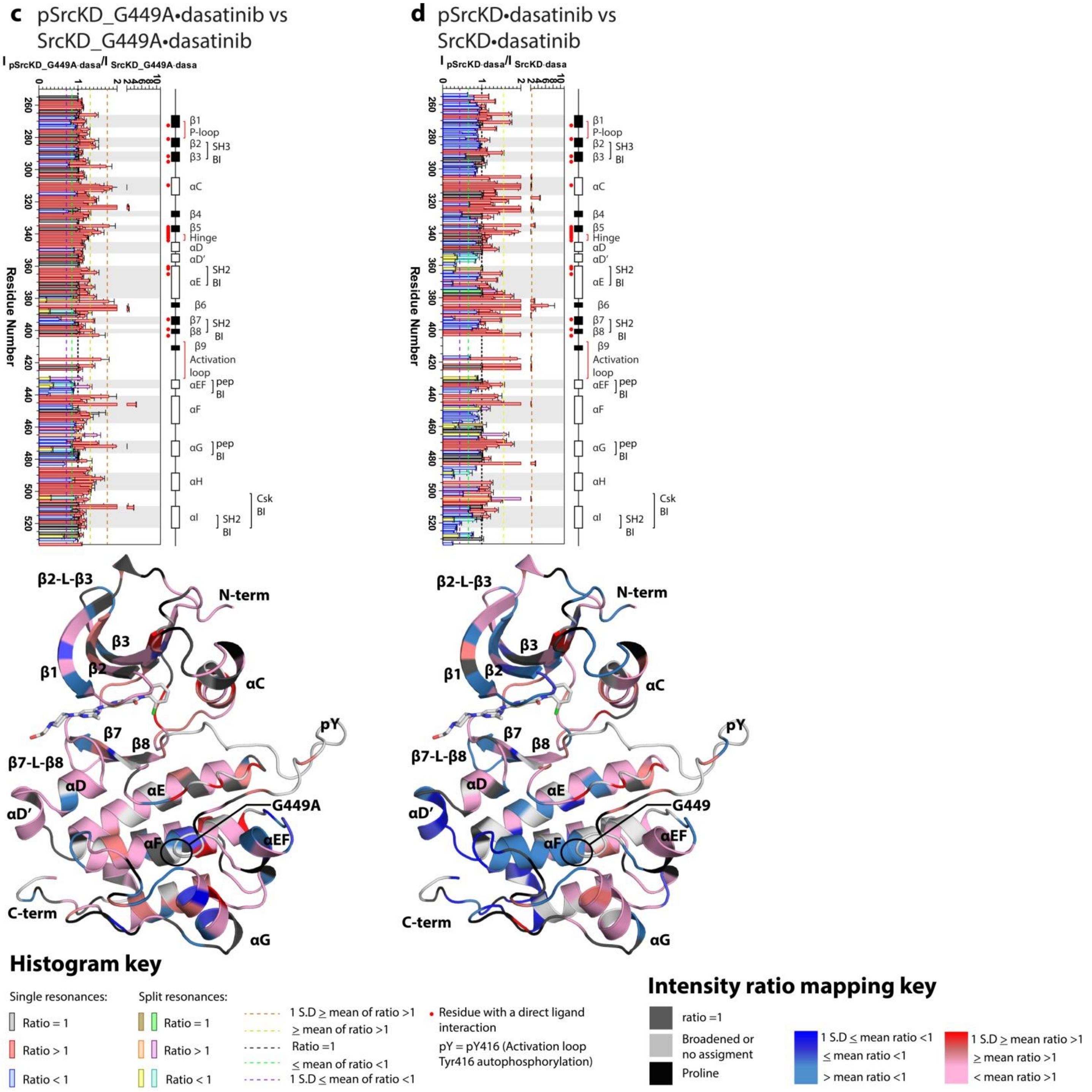

Supplementary Figure 16 | G449A induces distinct IPs in SrcKD in unphosphorylated and phosphorylated activation loop states. Intensity ratios between G449A and wild type in the context of **(a)** SrcKD•dasatinib and **(b)** pSrcKD•dasatinib were compared to probe the effect of the mutation on the dynamics of SrcKD in unphosphorylated and phosphorylated states. G449A mostly shows broadening throughout SrcKD•dasatinib. However, G449A in pSrcKD•dasatinib shows distinct regions undergoing pronounced resonance sharpening: helices-αD', αF, αI, and αG-αH loop, and carboxy-terminal tail. **(c)** Intensity ratios between pSrcKD_G449A•dasatinib and SrcKD_G449A•dasatinib were also analyzed to probe the effect of activation loop autophosphorylation on SrcKD dynamics in

the context of G449A and similarly for **(d)** wild type. Activation loop autophosphorylation in the mutant state induces more extensive resonance sharpening than wild type. Notably sharpening occurs across helix-αD' and the carboxy-terminal tail which contrasts with wild type, suggesting distinct modulation of the SH2/CsK binding interface.

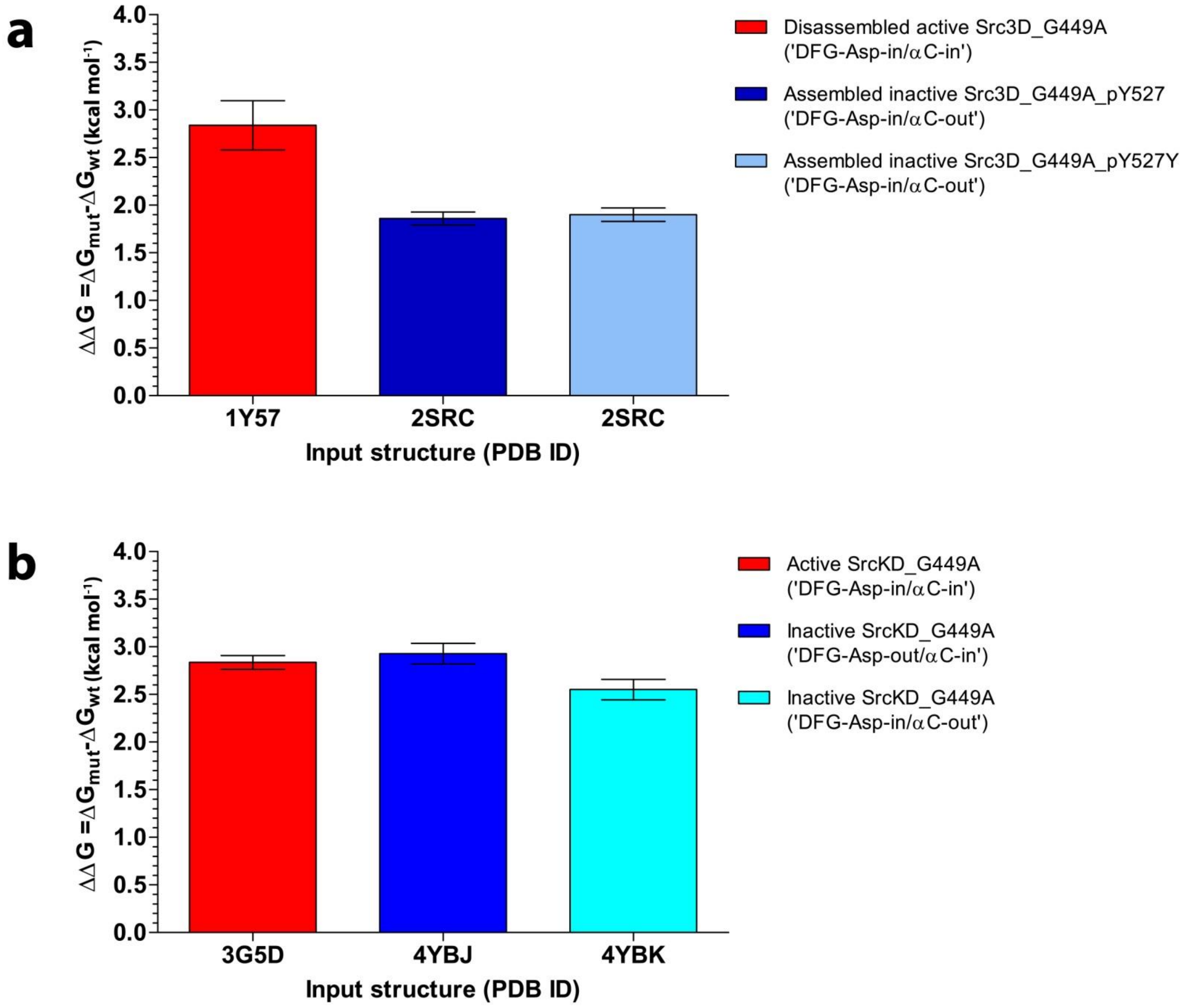

Supplementary Figure 17 | G449A differentially destabilizes different Src3D conformations but not SrcKD conformations, showing greater stability for the assembled inactive conformation over the disassembled active conformation. The change in free energy of stability (ΔΔG = ΔΔGmut-ΔGwt (kcal mol^{-1})) for **(a)** Src3D and **(b)** SrcKD in distinct active and inactive conformations upon introducing the G449A mutation were calculated using Foldx[4]. The disassembled active conformation is the most destabilized state. The assembled inactive conformations plus or minus pY527 (pY527Y mutant to mimic dephosphorylation of the carboxy-terminal tail) are similar in terms of destabilization suggesting that pY527 has minimal effect on counteracting the G449A destabilizing effect, but both are less destabilized than the assembled active state. The different SrcKD conformations all exhibit similar magnitudes of destabilization as a result of G449A. This suggests that the regulatory domains are implicated in reducing the overall destabilizing effect of G449A in the assembled inactive conformation.

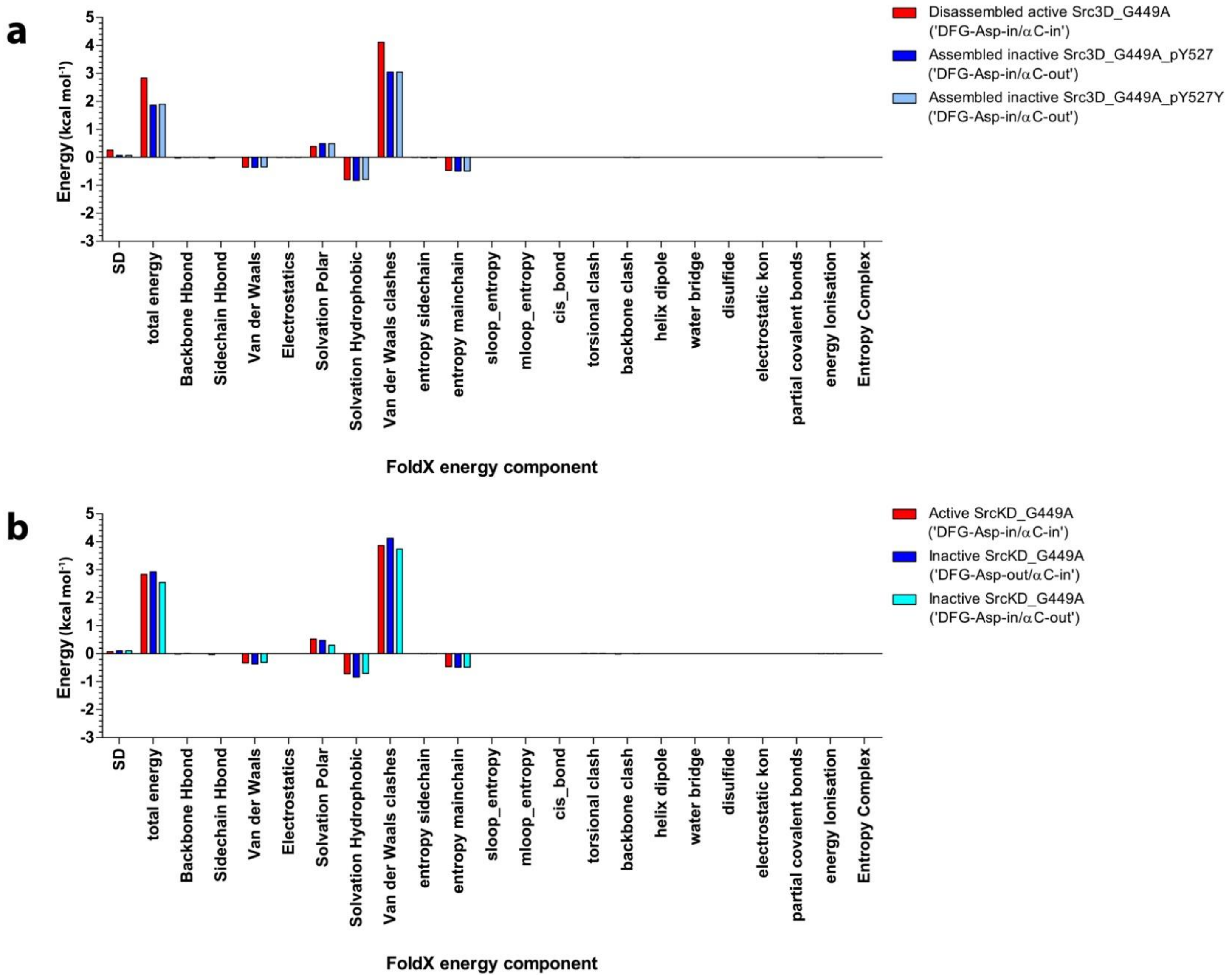

Supplementary Figure 18 | Van der Waal clashes are the main origin of the G449A destabilizing effect in Src3D and SrcKD conformations. Histogram plots of the Foldx energy components which contribute to the overall total energy ($\Delta\Delta G = \Delta\Delta G_{mut} - \Delta G_{wt}$ (kcal mol^{-1})) indicative of the change in stability for **(a)** Src3D conformations and **(b)** SrcKD conformations. Vdw clases give rise to the largest positive energy increase out of all the energy components in all conformations of Src3D and SrcKD. The magnitude of the vdw clash positive energy is greatest for the assembled active Src3D_G449A conformation, indicating that in this conformation the vdw clash is most severe.

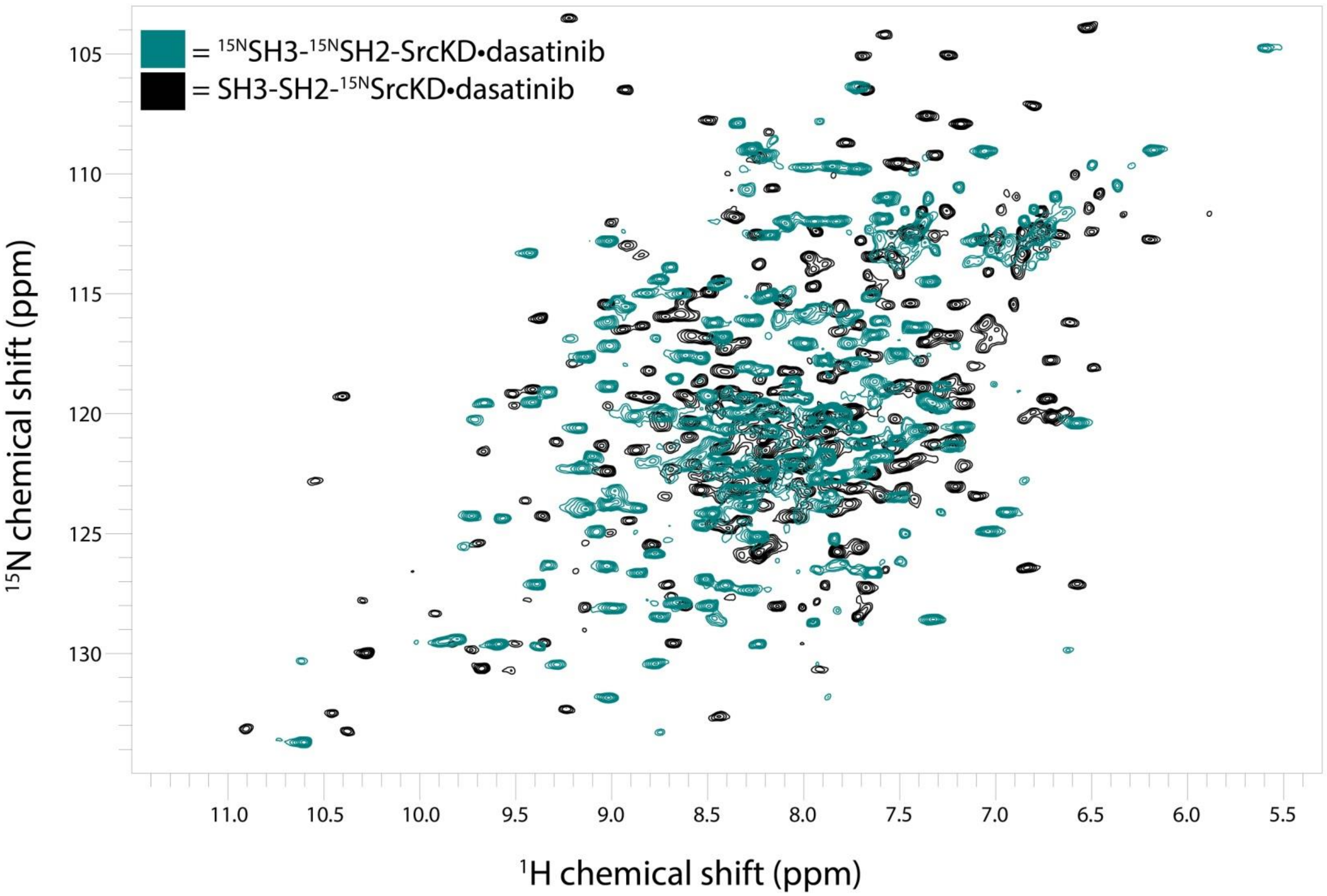

Supplementary Figure 19 | ^{1}H-^{15}N TROSY HSQC spectra of two Src3D fusion constructs with the catalytic SrcKD ^{15}N labeled and regulatory SH3-SH2 domains unlabeled, and vice versa. The spectra shows the complexity of spectral crowding that would be expected if a uniformly ^{15}N labeled Src3D sample were used .

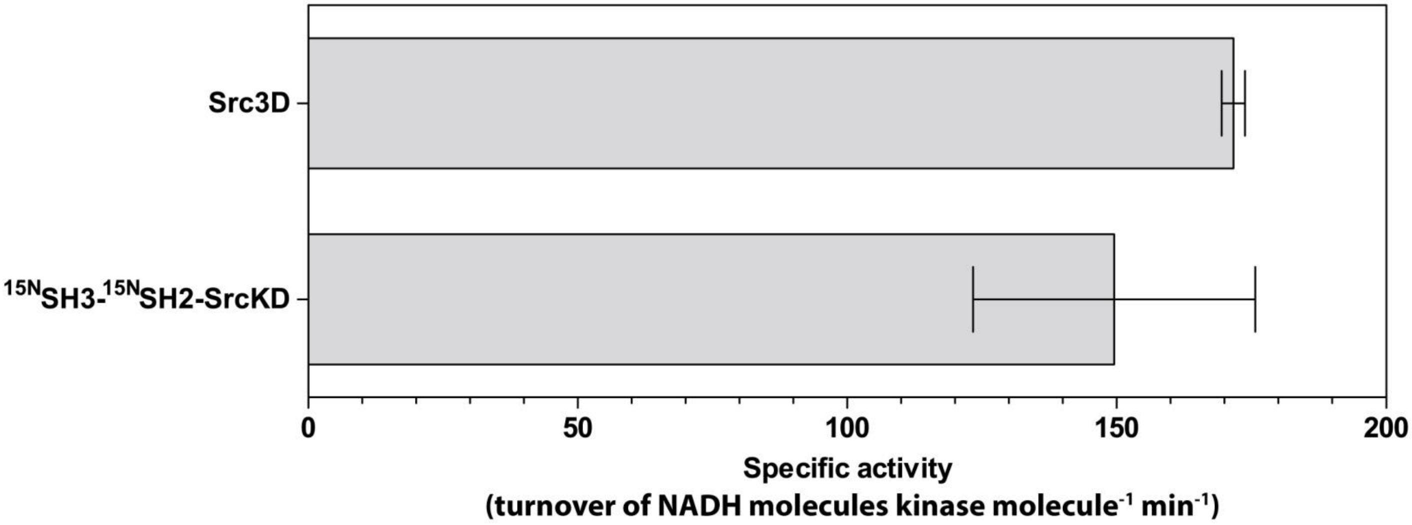

Supplementary Figure 20 | The Src3D fusion construct retains wild type like kinase activity. The specific activity of ^{15N}SH3-^{15N}SH2-KD is comparable to Src3D wild type. This indicates that sortase recognition motifs engineered at the SH3-SH2 carboxy-terminus and amino-terminus of SrcKD, and their subsequent ligation to generate Src3D fusion can reconstitute basal kinase activity that is similar to Src3D wild type.

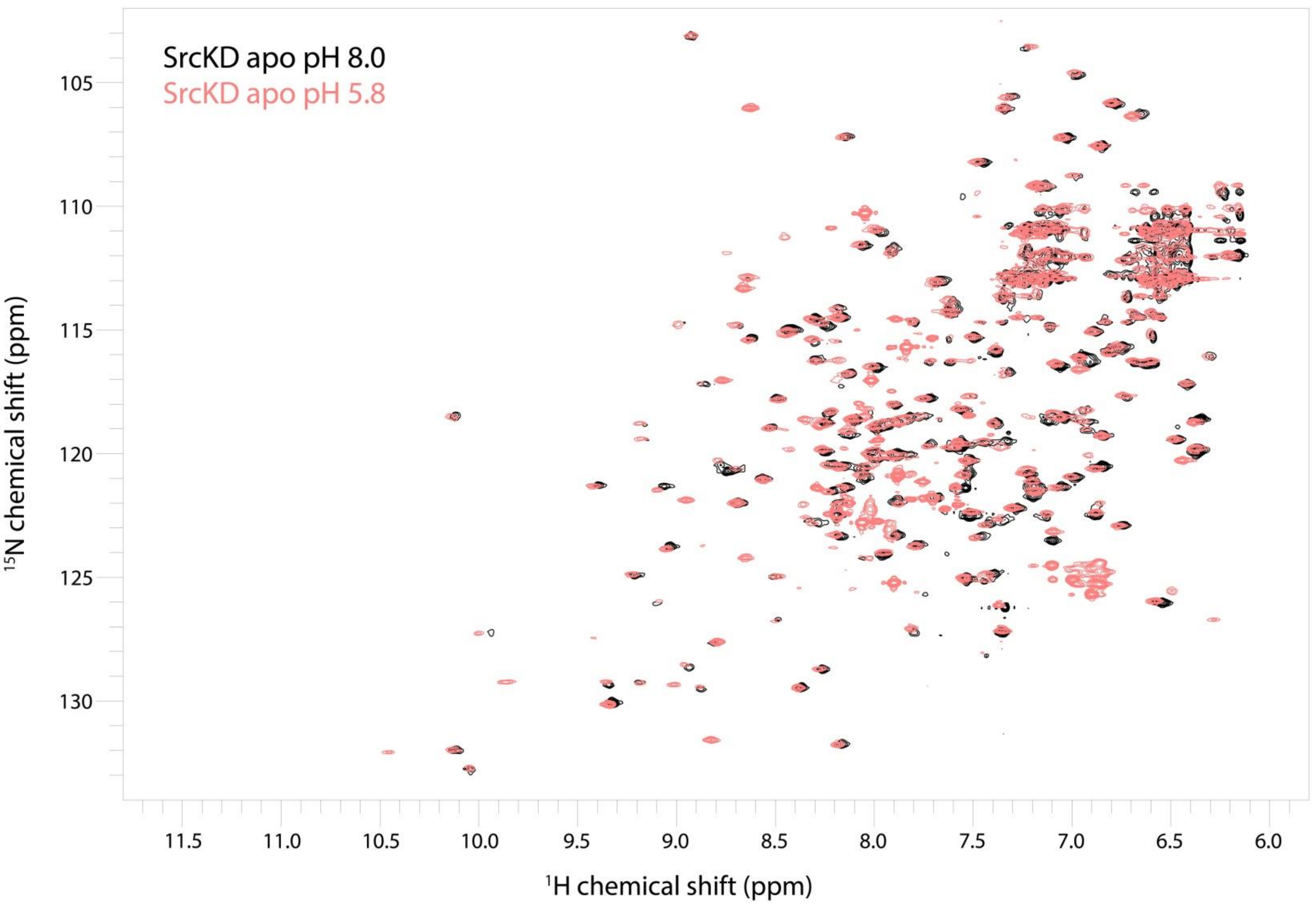

Supplementary Figure 21 | Superimposition of ^{1}H-^{15}N TROSY HSQC spectra at pH 8 and pH 5.8. More backbone amide resonances are observable at pH 5.8 compared to pH 8 because the resonances which are unobservable at pH 8 are in exchange with the bulk solvent, but at lower pH the exchange process is reduced.

Ligand	State	DFG-Asp-motif	Helix-αC	Affinity to Src
Dasatinib	Active	In	In	0.07±0.01 nM [1]
DAS-DFGO2	Inactive	Out	In	2.7±0.01 nM [1]
DAS-CHO2	Active	In	Out	1.7±0.5 nM [1]

Supplementary Table 1 | Panel of conformation selective ligands and their target conformations and binding affinities.

	Concentration [µM]	Amide Scaling Factor	Apparent T_2 (ms)	Apparent τc (ns)	Apparent Mw (kDa)	Methyl Scaling Factor	Apparent T_2 (ms)	Apparent τc (ns)	Apparent Mw (kDa)	Mean Apparent Mw (kDa)	Expected Mw (kDa)	Mw difference (kDa)
**SrcKD Apo**	180	1.613	10	19.1	38	1.582	11	18.3	37	37.5	33	4.5
**pSrcKD Apo**	180	1.609	11	19.0	38	1.613	10	19.1	38	38	33	5
**SrcKD•dasa**	180	1.613	10	19.1	38	1.597	11	18.7	37	37.5	33	4.5
**pSrcKD•dasa**	350	1.597	11	18.7	37	1.567	11	18.0	36	37	33	4.0
**pSrcKD•dasa•pep**	180	1.628	10	19.5	39	1.597	11	18.7	37	38	34.5	3.5
**Ubiquitin**	1000	1.192	28	7.0	14	1.192	28	7.0	14	14	9.5	4.5
**CyclophilinF**	460	1.314	18	10.9	22	1.327	18	11.3	23	22.5	18	4.5

Supplementary Table 2 | Two-point jump-and-return spin echo delay experiment parameters

pSrcKD	pSrcKD•dasatinib	pSrcKD•dasatinib•peptide	pSrcKD_G449A•dasatinib
319	-	-	-
-	354	354	-
355	355	355	-
357	357	357	-
362	362	362	-
376	376	376	-
-	-	-	381
-	-	-	388
430	430	430	430
-	-	-	434
-	-	-	435
449	449	449	-
459	459	459	-
460	460	-	-
465	465	-	465
-	-	-	474
489	489	489	-
-	-	-	504
505	505	-	-
518	518	518	-
528	528	-	528

Supplementary Table 3 | Residues with resonance splitting phenomena upon activation loop autophosphorylation of distinct SrcKD states

**Trimmed mean T_1 (s)**	**Trimmed mean T_2 (ms)**	**Mean global correlation time τc (ns)**	**Hydronmr calculated correlation time τc (ns)**
2.28	27.02	20.65	21.5

Supplementary Table 4 | Summary of NMR and Hydronmr derived dynamic parameters

	**K_m peptide (at 400 μM ATP)**	**K_m ATP (at 800 μM peptide)**
**SrcKD**	361 ± 25 μM	202 ± 32 μM
**SrcKD_G449A**	307 ± 25 μM	245 ± 18 μM
**Src3D**	>500 ± 287 μM	157 ± 51 μM
**Src3D_G449A**	>500 ± 118 μM	177 ± 20 μM

Supplementary Table 5 | The K_m for substrate peptide and K_m for ATP for Src3D and Src3D_G449A mutants are similar suggesting that the activity defect is not attributed to different substrate affinities. The activity defect towards substrate peptide is only observed in Src3D_G449A where the regulatory domains are present. Comparison of the K_m for substrate peptide for Src3D versus Src3D_G449A shows that both are greater than 500 μM, whilst for their K_m ATP there is only a 1.1-fold difference. Similarly G449A has minimal effect on the affinity of SrcKD for its substrates.

Supplementary Methods

NMR backbone assignment experiments

Triple resonance experiments HNCO, HNCACO, HN(CO)CA, HNCA, HN(COCA)CB, HNCACB[5, 6], were acquired on a triple labeled (^{2}H, ^{15}N, ^{13}C) pSrcKD•dasatinib sample at ~200 µM in 50 mM MES pH 5.8, 250 mM NaCl, 1 mM TCEP, 10% $^{2}H_2O$ at 30 °C. Backbone amide resonance assignments for this state was achieved for 239 residues out of 265 non-proline residues, corresponding to 90% of the Src kinase domain, which is defined by residues 251-533. Except for the partial assignments in the catalytic loop, DFG motif, and activation loop, all of the other key archetypal functional and regulatory elements are completely assigned. The lack of complete assignments for the activation loop coincides with the SrcKD•dasatinib crystal structure (PDB: 3G5D) in which the electron density was not fully resolved[2]. Presumably resonances for the DFG motif and activation loop were compromised beyond detection because of intermediate conformational exchange and not fast exchange with the solvent. The former scenario was more likely because solvent exposed residues in other parts of the kinase domain that were unobservable at pH 8.0, became observable at pH 5.8 (Supplementary Fig. 21), whereas the DFG motif and activation loop resonances remained unobservable at both pH. To validate our assignments, TALOS was used to calculate the secondary structure based on chemical shift information[7], and showed consistency with the secondary structure from the SrcKD•dasatinib crystal structure (PDB: 3G5D)[2]. Assignments were also transferred to the other states.

Molecular weight evaluation and NMR sample optimization

To ensure that the CSPs and IPs observed in SrcKD could be attributed to input signals affecting SrcKD, it was important for the apo and ligand bound states to behave as a monomer in the NMR experiments. Conditions under which the protein behaved as a monomer were evaluated by using a modified version of the Bruker p11 pulse sequence that implements 1-1 water suppression followed by a spin echo. This experiment involves a two point estimate of the non-sharp amide and methyl ^{1}H T_2 values by using one short (deltaA) and one long delay time (deltaB), to allow T_2 relaxation[8]. The scaling factor (scalef) between the spectra relates to the T_2, correlation time (tc), and molecular weight (mw [kDa]) of the protein sample by using the following formula[9]:

if scaling factor > 1.0, then, T_2 = 2 * (deltaA - deltaB) / log(scalef);
else, T_2 = 2 * (deltaB - deltaA) / log(scalef);
tc = 1.0 / (5.0 * T_2 * 0.001);

$mw = 2.0 * tc$

The method was validated using ubiquitin and CypF (Cyclophilin F) as controls, because these proteins behave as monomers. We confirmed that the samples were not aggregating under our buffer conditions at SrcKD concentrations ≥180 <200 µM, and between 200-400 µM, and that samples were more stable in solution at pH 6.4 compared to pH 5.8. Experiments were conducted at 30 °C with the delay times set to 0.4 and 2.9 ms.

IP experiments to infer dynamic changes

IPs calculated as the ratio of normalized intensities for two states (i.e. I_{ligand}/I_{apo}) are a proxy for dynamic changes in backbone amide resonances upon perturbation by i.e. ligand binding, autophopshorylation, mutagenesis. The magnitude of the intensity ratio reflects the contributions of two dynamic processes: (i) Fast pico- to nano second internal motions, described by the local effective correlation time (τ_e), promote resonance sharpening; (ii) Slow micro- to millisecond internal motions, described by the conformational exchange component R_{ex}, which enhances apparent R_2 relaxation, promote resonance broadening[10]. Thus, an increase in the conformational exchange contribution relative to the fast internal motion will result in an intensity ratio < 1 (broadening). An increase in the fast internal motion contribution relative to conformational exchange will result in an intensity ratio > 1 (sharpening). Thus, perturbations which cause the intensity ratio for a backbone resonance to deviate from a value of 1 imply such dynamic changes. A ratio of 1 suggests no change in conformational exchange and fast internal motion contributions or that their differential contributions cancel each other out so there is no relative change. Broadening and sharpening can also be attributed to an increase (slow tumbling) or decrease (fast tumbling) in the global correlation time (τ_c). Because τ_c is dependent on the molecular weight and hydrodynamic radius of the protein, resonance intensities are sensitive to aggregation/oligomerisation. However, we find that the global correlation time τ_c of SrcKD corresponds to that of a monomer under all experimental conditions tested here (Supplementary Fig. 3, Supplementary Table 2).

Backbone relaxation experiments

T_1 relaxation delays were set to 0.01, 0.05, 0.2, 1, 1.5, 2, 2.5, 3, 4 s, whereas T_2 relaxation delays were set to 8, 16, 24, 32, 40, 48, 56, 64 ms. All delay times were collected in duplicate. The T_1 and T_2 datasets were fitted to monoexponential decay curves described by $I(t) = I(t0) \times e(-t/T_1)$, and $I(t)$ =

I(t0) x e(-t/T_2), where I is intensity, in order to determine the T_1, T_2 time constants. Errors for the T_1, T_2 time constants were derived from the error associated with nonlinear fitting. A relaxation delay of 5 s was used for the (^{1}H)-^{15}N heteronuclear NOE unsaturated and saturated experiments. NOE ratios were determined in which the NOE ratio for each resonance = saturated I/unsaturated I. Errors in the (^{1}H)-^{15}N Heteronuclear NOE experiment were calculated through error propagation using the root mean square noise of the spectra. All spectra were processed using Topspin, with resonance intensities picked, analyzed, and fitted accordingly using CcpNmr Analysis. Backbone amide order parameters S^2 were determined using the RCI server[11]. Model-free formalism from ^{15}N relaxation data was performed using Fast-modelfree[12] and modelfree4[13], and their initial inputs were determined from pdbinteria, r2r1_tm, and quadric diffusion. Residues which deviated from the mean R_2:R_1 ratio by >1.5 S.D, or had large amplitude fast internal motions (NOE values <0.6) were excluded from the input residues used in quadric diffusion.

Hydronmr7c calculations

The τ_c for pSrcKD•dasatinib and the diffusion tensor model determined from FAST-modelfree was validated using hydronmr7c[14, 15] with the input PDB model 3G5D[2].

Foldx calculations

The effect of the G449A mutation on the stability of different Src structures was determined by using Foldx[4] to calculate the change in free energy of stability (ΔΔG = ΔΔGmut-ΔGwt (kcal mol^{-1})). The input PDBs used were: 1Y57, 2SRC, 3G5D, 4YBJ, 4YBK[1, 2, 16]. Foldx error bars were based on the s.d of performing three runs.

Supplementary References

1. Kwarcinski FE*, et al.* Conformation-Selective Analogues of Dasatinib Reveal Insight into Kinase Inhibitor Binding and Selectivity. *Acs Chem Biol* **11**, 1296-1304 (2016).

2. Getlik M*, et al.* Hybrid compound design to overcome the gatekeeper T338M mutation in cSrc. *J Med Chem* **52**, 3915-3926 (2009).

3. Wheeler TJ, Clements J, Finn RD. Skylign: a tool for creating informative, interactive logos representing sequence alignments and profile hidden Markov models. *BMC bioinformatics* **15**, 7 (2014).

4. Guerois R, Nielsen JE, Serrano L. Predicting changes in the stability of proteins and protein complexes: a study of more than 1000 mutations. *J Mol Biol* **320**, 369-387 (2002).

5. Yamazaki T, Lee W, Arrowsmith CH, Muhandiram D, Kay LE. A suite of triple resonance NMR experiments for the backbone assignment of 15N, 13C, 2H labeled proteins with high sensitivity. *Journal of the American Chemical Society* **116**, 11655-11666 (1994).

6. Shan X, Gardner KH, Muhandiram D, Rao N, Arrowsmith CH, Kay LE. Assignment of 15N, 13Cα, 13Cβ, and HN Resonances in an 15N, 13C, 2H Labeled 64 kDa Trp Repressor− Operator Complex Using Triple-Resonance NMR Spectroscopy and 2H-Decoupling. *Journal of the American Chemical Society* **118**, 6570-6579 (1996).

7. Shen Y, Delaglio F, Cornilescu G, Bax A. TALOS+: a hybrid method for predicting protein backbone torsion angles from NMR chemical shifts. *J Biomol NMR* **44**, 213-223 (2009).

8. Sklenář V, Bax A. Spin-echo water suppression for the generation of pure-phase two-dimensional NMR spectra. *Journal of Magnetic Resonance (1969)* **74**, 469-479 (1987).

9. Anglister J, Grzesiek S, Ren H, Klee CB, Bax A. Isotope-edited multidimensional NMR of calcineurin B in the presence of the non-deuterated detergent CHAPS. *J Biomol NMR* **3**, 121-126 (1993).

10. Brettmann JB, Urusova D, Tonelli M, Silva JR, Henzler-Wildman KA. Role of protein dynamics in ion selectivity and allosteric coupling in the NaK channel. *Proc Natl Acad Sci U S A* **112**, 15366-15371 (2015).

11. Berjanskii MV, Wishart DS. Application of the random coil index to studying protein flexibility. *J Biomol NMR* **40**, 31-48 (2008).

12. Cole R, Loria JP. FAST-Modelfree: a program for rapid automated analysis of solution NMR spin-relaxation data. *J Biomol NMR* **26**, 203-213 (2003).

13. Mandel AM, Akke M, Palmer AG, 3rd. Backbone dynamics of Escherichia coli ribonuclease HI: correlations with structure and function in an active enzyme. *J Mol Biol* **246**, 144-163 (1995).

14. Garcia de la Torre J, Huertas ML, Carrasco B. HYDRONMR: prediction of NMR relaxation of globular proteins from atomic-level structures and hydrodynamic calculations. *J Magn Reson* **147**, 138-146 (2000).

15. Bernado P, Garcia de la Torre J, Pons M. Interpretation of 15N NMR relaxation data of globular proteins using hydrodynamic calculations with HYDRONMR. *J Biomol NMR* **23**, 139-150 (2002).

16. Cowan-Jacob SW*, et al.* The crystal structure of a c-Src complex in an active conformation suggests possible steps in c-Src activation. *Structure* **13**, 861-871 (2005).
